# Directional Exosome Proteomes Reflect Polarity-Specific Functions in Retinal Pigmented Epithelium Monolayers

**DOI:** 10.1038/s41598-017-05102-9

**Published:** 2017-07-07

**Authors:** Mikael Klingeborn, W. Michael Dismuke, Nikolai P. Skiba, Una Kelly, W. Daniel Stamer, Catherine Bowes Rickman

**Affiliations:** 1 Department of Ophthalmology, Duke Eye Center, Duke University, Durham, NC 27710 USA; 2 Department of Biomedical Engineering, Duke University, Durham, NC 27710 USA; 3 Department of Cell Biology, Duke University, Durham, NC 27710 USA

## Abstract

The retinal pigmented epithelium (RPE) forms the outer blood-retinal barrier in the eye and its polarity is responsible for directional secretion and uptake of proteins, lipoprotein particles and extracellular vesicles (EVs). Such a secretional division dictates directed interactions between the systemic circulation (basolateral) and the retina (apical). Our goal is to define the polarized proteomes and physical characteristics of EVs released from the RPE. Primary cultures of porcine RPE cells were differentiated into polarized RPE monolayers on permeable supports. EVs were isolated from media bathing either apical or basolateral RPE surfaces, and two subpopulations of small EVs including exosomes, and dense EVs, were purified and processed for proteomic profiling. In parallel, EV size distribution and concentration were determined. Using protein correlation profiling mass spectrometry, a total of 631 proteins were identified in exosome preparations, 299 of which were uniquely released apically, and 94 uniquely released basolaterally. Selected proteins were validated by Western blot. The proteomes of these exosome and dense EVs preparations suggest that epithelial polarity impacts directional release. These data serve as a foundation for comparative studies aimed at elucidating the role of exosomes in the molecular pathophysiology of retinal diseases and help identify potential therapeutic targets and biomarkers.

## Introduction

The retinal pigmented epithelium (RPE) is a cell monolayer that is situated between the photoreceptors and the systemic circulation of the choroid. The RPE is the initial site of pathological changes in age-related macular degeneration (AMD), which is the leading cause of blindness in people 65 years of age or older in developed countries^1^. RPE cells are highly specialized and active phagocytic cells that carry out crucial functions in the eye, such as daily phagocytosis of outer segments shed from rod and cone photoreceptors, processing and transport of nutrients, and recycling of visual pigments^2^. The RPE forms the outer blood-retinal barrier in the eye and its polarity is responsible for the directional secretion of proteins, lipoprotein particles and lipid bilayer-enclosed extracellular vesicles (EVs). Such polarity dictates directed interactions between the systemic circulation (basolateral) and the retina (apical). RPE cells take up lipoprotein particles at their basolateral surface from the systemic circulation, repackage lipids and lipoproteins into new lipoprotein particles which are then delivered from its apical surface to photoreceptors^3–5^. Waste products and lipoprotein particles from the photoreceptors are in turn trafficked back to the RPE for recycling and removal^4–6^. The role of this extensive endocytic trafficking, including the formation and release of a range of EVs, in AMD and other retinal diseases has not been thoroughly investigated to date^7^.

Exosomes are cell-derived, bilayer-enclosed, nanovesicles (ø = 30–150 nm) that are secreted in a controlled manner from most cell types. They make up the smallest subpopulation of the wide range of EVs released from most cells. It has become increasingly clear in recent years that exosomes have specialized functions and play a key role in, among other things, intercellular signaling, and cellular waste management^8^. The results from a number of studies suggest that exosomes are not secreted merely as a degradation route for redundant molecules^9^; rather they are equipped to withstand lysis by the complement system to carry out extracellular functions^10^. Exosomes are formed inside a specialized endosome called a multivesicular endosome (MVE) and are released into the extracellular milieu upon MVE fusion with the plasma membrane. Their biogenesis and extracellular release is distinct from other EVs such as larger ectosomes that bud directly from the plasma membrane^11^. Exosomes and ectosomes are also functionally distinct in many respects^11^. The role of exosomes and other EVs in the healthy and diseased eye has only recently begun to undergo rigorous study (reviewed in ref. 7). Polarized cells such as epithelia, neurons and lymphocytes, have in some cases been shown, and in other cases hypothesized, to release exosomes in a directional manner with different cargoes in apical versus basolateral exosomes^12–15^. However, there is a paucity of these studies to date, and none have used a global approach to characterize the protein exosome content in its entirety.

Cells under stress are known to increase the release of membranous vesicles including exosomes^16^, and this has also been suggested to be the case in RPE cells^17^. Interestingly, the mainly apical exosomal release of the heat shock protein αB-Crystallin from polarized RPE cultures, was shown to be altered to a bidirectional release when the cells experienced stress conditions^15^, suggesting a potential protective exosomal response. Studies have shown that exosomes released by stressed RPE exhibit changes in signaling phosphoproteins^18^, and are coated with complement components^19, 20^, including the terminal membrane attack complex, C5b-9^21^. Furthermore, a recent *in vitro* study found that small EVs released from cultures of the spontaneously immortalized RPE cell line ARPE-19, promoted an immunoregulatory phenotype in monocytes^22^. Thus, RPE-derived exosomes may affect both innate and cellular immune functions in the outer retina and the Bruch’s membrane-choroid complex. Additionally, proteins found in the sub-RPE deposits or drusen, associated with AMD contain proteins such as enolase and ATP synthase^23^, two proteins commonly found in exosomes^24^, supporting an exosomal origin for some drusen components. Interestingly, a study by Ebrahimi and co-workers suggested that decreased levels of the complement regulators CD46 and CD59 on RPE cells during the AMD disease process, were in part explained by their release in exosomal and apoptotic membranous particles. It was suggested that this decrease of membrane complement regulators on RPE cells were in part responsible for inadequate control of complement by the RPE in AMD, which induces RPE damage^25^. Although these studies support a role for RPE-derived exosomes in outer retinal health and disease, they were limited by one or more factors. For example often the studies were not conducted using *bona fide* RPE cell cultures (*i*.*e*. ARPE-19 is an RPE-*like* cell line, lacking several important hallmarks of RPE cells)^26, 27^, and the exosome characterization was limited, lacking detail and newly established exosome markers^17–20^. Most important, the majority of previous studies did not use highly polarized RPE cultures grown on permeable supports (*i*.*e*. Transwell, ThinCert, or Millicell)^17–20, 22^. The use of primary cultures of polarized RPE cell monolayers grown on semi-permeable membrane supports is essential to study basolateral-specific cellular functions in addition to the commonly studied apical functions.

Finally, previous studies were limited because they relied on traditional mass spectrometric analysis of exosome and small EV preparations, which are inherently heterogeneous mixtures, and cannot easily identify protein components of low abundance that are nonetheless specific for exosomes and small EVs. Moreover, highly abundant proteins are often contaminants, but may be difficult to recognize as such. To overcome these technical issues, we performed a mass spectrometry-based proteomic analysis of apically and basolaterally RPE-derived EVs by simultaneously profiling hundreds of proteins in EV preparations of increasing purity. This approach, termed Protein Correlation Profiling (PCP)^28, 29^, permits the analysis of any sub- or extracellular components/complexes that can be enriched by fractionation but not purified to homogeneity. Such a stepwise paired analysis by PCP provides a powerful approach to both identify *bona fide* resident proteins and to exclude contaminating proteins from a proteomic dataset, allowing for identification of highly enriched proteins of high as well as very low abundance in the pure exosome preparations. This is the first study to use PCP in determining exosome proteomes.

The ability to conduct studies of the potential role of EVs in the pathophysiology of AMD and other retinal diseases affecting the RPE relies on having quality baseline data for physiologically normal RPE-derived EV release. Thus, in the present study we determined the proteomic content, size distribution and concentration of highly purified extracellular vesicles released apically and basolaterally from a differentiated polarized primary porcine RPE cell culture model.

## Results

### Characterization of polarized primary porcine retinal pigmented epithelium monolayers

**Figure 1:**
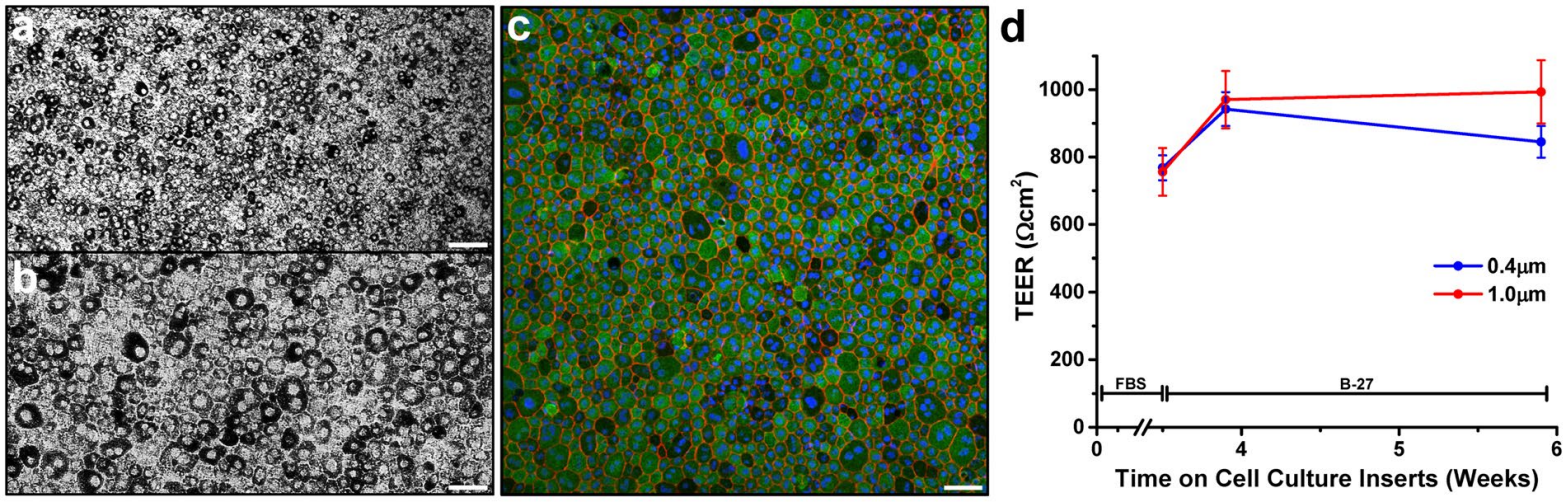
Morphology and barrier function of primary cultures of porcine RPE (pRPE) monolayers. (**a–c**) pRPE cultures grown for three weeks on 0.4 µm pore size cell culture inserts in B-27 supplemented media. (**a**,**b**) Light micrographs using 10X (**a**) and 20X objectives (**b**) show high level of pigmentation. (**c**) Confocal immunofluorescence microscopy of F-actin with phalloidin staining (red) along cell borders highlight characteristic hexagonal cell shape. Widespread cytosolic immunoreactivity for RPE65 (green), a specific metabolic marker of RPE, indicates highly differentiated RPE cells. Nuclei were counterstained with Hoechst dye (blue). Scale bars are 100 µm (**a**) and 50 µm (**b**,**c**). (**d**) To assess the integrity of pRPE monolayers grown in media supplemented with B-27 serum supplement, the transepithelial electrical resistance (TEER) was measured first in cells on inserts for 3.5 weeks in medium supplemented with 1% FBS and then on cells where medium was replaced with 2% B-27 serum supplement. TEER remained elevated on cells grown on inserts with two different pores sizes for the duration of collection of conditioned B-27 supplemented media. Values plotted are Mean ± S.E.M., six to fifteen replicates per data point.

This study was carried out using mature, polarized primary porcine RPE (pRPE) cultures grown on transwell inserts for up to 6 weeks. Primary cultures of pRPE cells were differentiated into polarized RPE monolayers on permeable supports. The morphology and state of differentiation of the RPE monolayer was assessed by immunofluorescence staining for RPE65, ZO-1 and cytokeratin. Micrographs showed robust pigmentation, immunoreactivity and correct cytosolic localization of RPE65 and cytokeratin, plus lateral cell distribution of ZO-1 (Figs 1a–c, S1 and S2). In addition, confocal immunofluorescence imaging for the RPE proteins Na^+^/K^+^-ATPase alpha (apical) and Bestrophin-1 (basal and lateral) known to have polarized localization in terminally differentiated but not in poorly differentiated RPE cells^30, 31^, revealed correct localization in our pRPE cultures (Fig. S3). In order to confirm that the pRPE cells expressed known RPE-specific markers, lysates of pRPE grown on transwells were analyzed by mass spectrometry. RPE-specific markers including RPE65, Bestrophin-1, CRALBP, RPB1, and a number of other visual cycle and retinoid metabolism proteins were identified (Supplemental Table S11). Apical microvilli and basal infoldings were also evident in pRPE cultures by electron microscopy imaging (Fig. S4c). Phalloidin staining revealed a normal hexagonal RPE cell shape in cultures grown on supports with the smallest pore size (0.4 µm). In contrast, we observed F-actin stress fibers and dysmorphic cell arrangements in cultures grown on the larger pore size supports (1.0 and 3.0 µm) (Fig. S1). In all the RPE cultures in which conditioned media was harvested for EV preparations we used a commercially available defined serum supplement (B-27) in order to avoid potential sources of contaminating particles from FBS as well as avoiding contamination of EV preparations with highly abundant soluble FBS proteins. To make sure that the epithelial cell monolayer barrier function was maintained in cultures with B-27-supplemented media versus FBS-supplemented media, the transepithelial electrical resistance (TEER) was measured 3 days (0.4 weeks) and 17 days (2.4 weeks) after cultures were switched from medium containing 1% FBS to medium supplemented with 2% B-27 (Fig. 1d). Seventeen days represented the longest time cultures were maintained in B-27 supplemented medium before use. There was an increase in the TEER following three days of B-27 supplementation compared to measurements immediately prior to the media change (0.4 µm: 768 ± 37 vs. 942 ± 50 Ωcm^2^; 1.0 µm: 756 ± 71 vs. 970 ± 85 Ωcm^2^), indicating intact tight junctions. After 17 days of B-27 conditions, TEER values (0.4 µm pore: 845 ± 47; 1.0 µm pore: 993 ± 94 Ωcm^2^) remained high, indicating that junctional interactions were maintained in the monolayers (Fig. 1d). The TEER in monolayers grown on the largest pore inserts (3.0 µm) were dramatically reduced suggesting that integrity of the tight junctions were compromised compared to monolayers grown on inserts with smaller pores (Fig. S4a) and in agreement with increased F-actin stress fiber staining (Fig. S1). Taken together these data show that our pRPE culture model exhibits important hallmarks of *bona fide* RPE cells.

### Physical characteristics of directionally released extracellular vesicles (EVs)

EVs were isolated from conditioned medium bathing either apical or basolateral RPE surfaces and analyzed. The size distribution and concentration of EVs released from both sides of the RPE cultures were determined by Nanoparticle Tracking Analysis (NTA). RPE cells maintained in B-27 supplemented medium released similar numbers of EVs on the apical and basolateral side (Table 1). Interestingly, RPE cells maintained in FBS-supplemented medium released approximately 4 to 15-fold (cells on 1.0 vs. 0.4 µm supports) less EVs on the basolateral side compared to the apical side on average (Table 1), although this did not reach statistical significance. No statistically significant differences between apical and basolateral EV concentration or size distribution were seen in the populations isolated from RPE cells grown on 0.4 or 1.0 µm inserts using either culture condition, except in one comparison (Tables 1 and 2).

**Table 1:** Concentration of extracellular vesicles released from porcine RPE cultured on permeable supports.

	Per well per 24 h (×10^6^ particles)	
	0.4 µm pore	1.0 µm pore
Apical – FBS	29.2 ± 9.6	12.4 ± 1.7
Basal – FBS	1.90 ± 0.22	3.25 ± 0.43
Apical – B-27	18.6 ± 3.1	35.3 ± 7.7
Basal – B-27	22.4 ± 4.2	34.4 ± 8.6

± = SEM.

**Figure 2:**
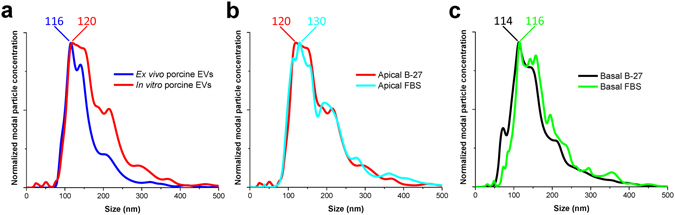
Size distribution of extracellular vesicles (EVs) released from polarized RPE monolayers. (**a**) EVs released from RPE in *ex vivo* porcine eyecups (blue trace) compared to those released apically from polarized RPE grown on cell culture inserts of 0.4 µm pore size (red trace). Note that the modal (most frequent) particle size is very similar (116.0 vs 120.0 nm) between the *ex vivo* and *in vitro* EV preparations. (**b**,**c**) The size distribution of EVs released apically (**b**) and basolaterally (**c**) from polarized RPE, are not statistically different in modal particle size (indicated in nm in graphs here and Table 2) under B-27- or FBS-supplemented culturing conditions. Size distributions displayed are averages of three or more separate experiments.

**Figure 3:**
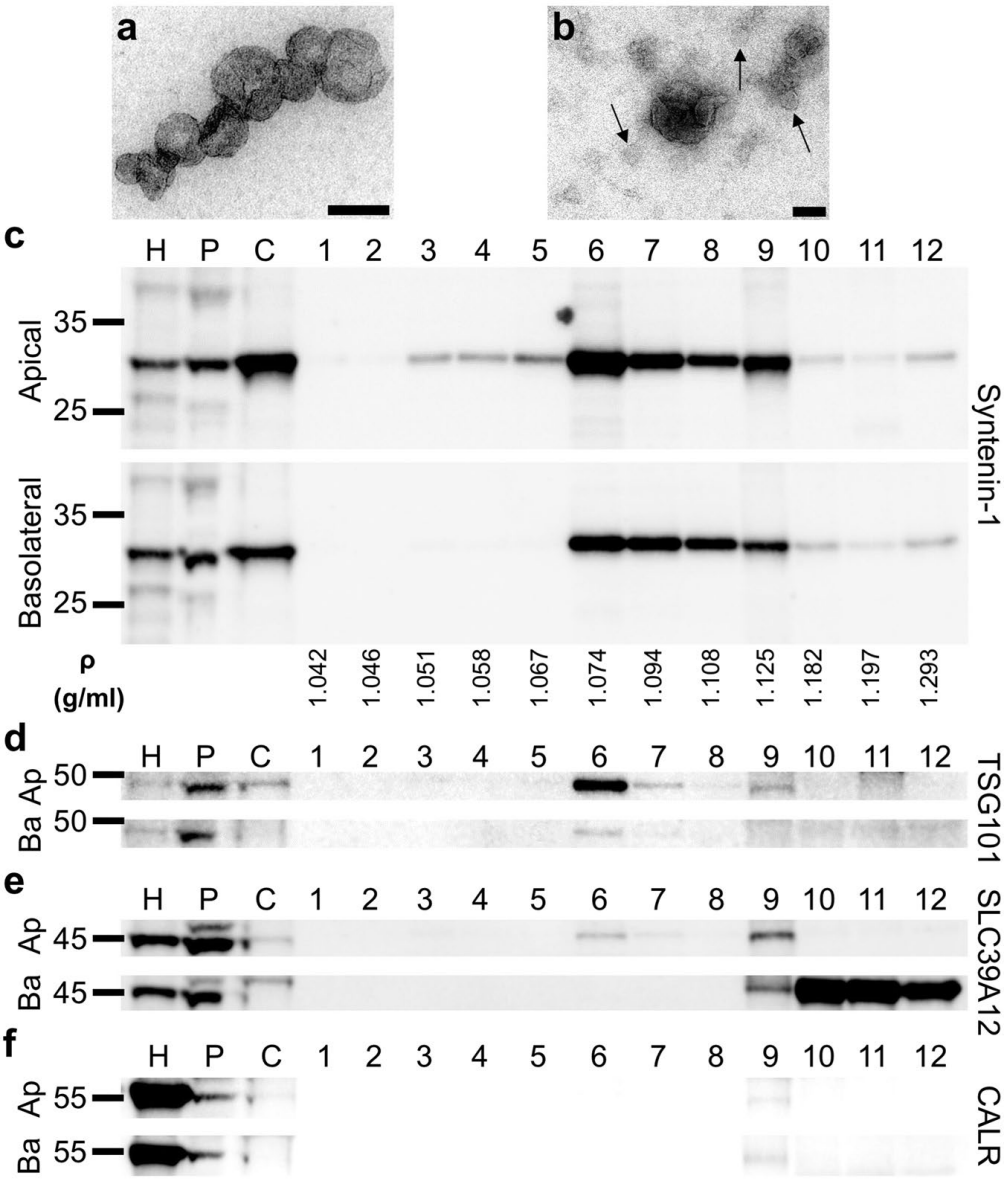
Electron microscopic and immunoblotting characterization of exosome preparations. (**a**,**b**) Electron micrographs of EVs in 100,000 *g* pellet. Vesicles of exosome sizes (30–150 nm) are seen in apical (**a**) and basolateral (**b**) EV preparations from polarized RPE cultures. A number of smaller vesicles (<50 nm) are indicated by arrows in panel (**b**). Scale bars 100 nm. (**c**–**f**) Representative immunoblots of apical and basolateral crude EV preparations and iodixanol density gradient fractions. Densities of fractions 6–8 correspond to the density of exosomes^34^. Immunoblots were probed with antibodies to the canonical exosome markers Syntenin-1 (**c**) or TSG101 (**d**). By mass spectrometry TSG101 is more abundant in apical exosome preparations than in basolateral, and this is also the case here by immunoblotting, see (**d**). (**e**) The zinc transporter SLC39A12 was only identified in the apical exosome proteome by PCP and is likewise only detected in apical exosome fractions by immunoblotting [Apical (Ap) panel, fractions 6–8]. (**f**) The ER marker Calreticulin (CALR) was not detected in exosome fractions 6–8, demonstrating that there was no contamination of ER fragments from apoptotic cells. A weak but detectable signal in fraction 9 is consistent with known presence of ER-resident proteins in dense EVs and/or ectosomes^34, 43^. H, human RPE-choroid lysate (20 µg); P, porcine polarized RPE lysate (20 µg); C, crude EV pellet (Apical: 13.1 µg, Basolateral: 16.6 µg); 1–12, fractions from top to bottom from OptiPrep density gradient; Ap, Apical; Ba, Basolateral. Apparent molecular weight markers and fraction densities are indicated.

The size distribution of EVs released apically from human eyecups (posterior half with vitreous and neuroretina removed and RPE exposed) *ex vivo* has recently been described^32^. Thus we analyzed EVs isolated from pig eyecups and EVs from our *in vitro* cultures to compare the size distribution of *ex vivo* pig RPE-derived EVs with our *in vitro* RPE-derived EVs (Fig. 2a). The modal (most frequent) particle size was very similar (116.0 vs. 120.0 nm) between the *ex vivo* and *in vitro* EV preparations, indicating that our *in vitro* RPE culture reproduced the EV release from RPE *in situ*. The size distribution of EVs released apically (Fig. 2b) or basolaterally (Fig. 2c) under B-27- and FBS-supplemented culture conditions, was also similar based on modal sizes (Student’s t-test; p > 0.05, thus no statistically significant difference, Table 2). In addition, there was no significant difference found between EVs released from RPE monolayers grown on 0.4 vs. 1.0 µm insert cultures (Table 2, Fig. S5). EV preparations were analyzed by electron microscopy and the majority of vesicles in both the apical (Fig. 3a) and the basolateral (Fig. 3b) EV preparations were the size of exosomes (30–150 nm) and displayed the traditional collapsed cup-shaped morphology of exosomes^33^.

**Table 2:** Modal and mean sizes of extracellular vesicles released from porcine RPE cultured on permeable supports.

	Cell culture insert pore size			
	0.4 µm		1.0 µm	
	Mode (nm)	Mean (nm)	Mode (nm)	Mean (nm)
Apical – FBS	118.6 ± 9.9	191.2 ± 7.8	100.7 ± 10.6	160.3 ± 8.2
Basal – FBS	132.2 ± 13.1	176.4 ± 8.7	108.7 ± 5.2	152.5 ± 3.8
Apical – B-27	125.9 ± 8.6	180.2 ± 7.9^*^	130.3 ± 16.7	163.0 ± 12.9
Basal – B-27	117.4 ± 12.5	146.5 ± 13.9	118.4 ± 11.8	151.1 ± 10.8

^*^p < 0.05 compared to 0.4 µm basal B-27 mean EV size; ±  = SEM.

Based on our characterization of RPE cultures and EV release on supports with different pore sizes, we used RPE cultures grown on 0.4 µm pore size supports for all experiments going forward.

### Isolation of EV populations highly enriched for exosomes

Iodixanol flotation density gradient centrifugation was used to specifically isolate exosomes and small EVs, which are known to equilibrate (float) in fractions in the density range of 1.07–1.11 g/ml^34^. Compared to the 100,000 *g* pellet of the cleared supernatant from a 2,000 *g* centrifugation (designated *crude EV pellet*), our gradient fractions with densities of 1.074–1.108 g/ml (fractions 6–8) were heavily enriched in small EVs and *bona fide* exosomes. Fractions were analyzed by immunblotting with two exosome markers, Syntenin-1 and TSG101^34^ to demonstrate exosome enrichment (Fig. 3c,d).

### Assessment of EV preparation purity and validation of polarity-specific exosome protein content

The high enrichment for known exosomal and small EV markers, and the lack of enrichment of known markers for ectosomes and/or large and dense EVs, in our exosome preparations (Tables 3–4) indicated a high level of purity of our preparations. Importantly, known contaminants of EV preparations such as apolipoproteins, were not enriched in these exosome preparations (Tables 3–4, S1–S4). To validate the polarity-specific nature of our exosome preparations, we also immunoblotted them for SLC39A12 which was only present in the proteome of apically released exosomes (Fig. 3e). Calreticulin, which is an ER-resident protein, was not detected in fractions 6–8 (Fig. 3f), demonstrating a lack of contamination due to ER fragments which are released during cell death. Taken together, these immunoblots show a high abundance of exosome markers, correct polarity-specific presence of proteins in iodixanol density fractions 6–8 (which were used to generate exosome preparations), and a lack of contamination with other organelles.

**Table 3:** Proteins enriched in apically released exosomes.

Enrichment ranking	Protein name	Gene name	Average Pure/crude^*^
1	CD81	CD81	1.07
2	Synaptosome Associated Protein 23	SNAP23	1.07
3	CD9	CD9	1.04
4	Syntenin-1	SDCBP	1.00
5	Hsp70-interacting protein	ST13	0.97
6	G Protein-Coupled Receptor Class C Group 5 Member C	GPRC5C	0.97
7	Prostaglandin F2 receptor negative regulator	PTGFRN	0.92
8	CD63	CD63	0.74
9	Neuronal membrane glycoprotein M6A	GPM6A	0.73
10	Integrin beta-5	ITGB5	0.72
11	14-3-3 Gamma	YWHAG	0.65
12	Heat shock 70 kDa protein 1B	HSPA1B	0.64
13	Copine 2	CPNE2	0.62
14	Brain Abundant Membrane Attached Signal Protein 1	BASP1	0.61
15	Solute Carrier Family 39 Member 12 (ZIP12)	SLC39A12	0.61
16	Solute Carrier Family 7 Member 5	SLC7A5	0.61
17	ALIX	PDCD6IP	0.59
18	14-3-3 Eta	YWHAH	0.59
19	CNDP Dipeptidase 2	CNDP2	0.59
20	Bone Marrow Stromal Cell Antigen 1	BST1	0.57
21	Annexin A11	ANXA11	0.57
22	Tumor susceptibility 101 protein	TSG101	0.55
23	Phosphoglycerate Mutase 1	PGAM1	0.52
24	CD82	CD82	0.50
25	Coxsackievirus and Adenovirus Receptor	CXADR	0.47
26	Heat Shock Protein Family A (Hsp70) Member 2	HSPA2	0.47
27	Radixin	RDX	0.46
28	Vesicle Associated Membrane Protein 3	VAMP3	0.46
29	Tweety Family Member 2	TTYH2	0.45
30	Solute Carrier Family 6 Member 8 (Creatine transporter)	SLC6A8	0.43
31	S100 calcium binding protein A14	S100A14	0.43
32	Ubiquitin C	RPS27A	0.41
33	Neprilysin	MME	0.41
34	Solute Carrier Family 6 Member 20	SLC6A20	0.41
35	Annexin A1	ANXA1	0.41
36	Transgelin	TAGLN	0.40
37	Epidermal Growth Factor	EGF	0.39
38	DnaJ homolog subfamily A member 2	DNAJA2	0.39
39	Glycoprotein M6B	GPM6B	0.39
40	Crystallin Alpha B	CRYAB	0.39
41	Ras Related GTP Binding Protein B	RALB	0.38
42	EGF-like Repeat and Discoidin I-Like Domain-Containing Protein 3	EDIL3	0.38
43	Phosphatidylethanolamine Binding Protein 1	PEBP1	0.38
44	5′-nucleotidase ecto	NT5E	0.37
45	Annexin A7	ANXA7	0.37
46	Annexin A2	ANXA2	0.37
47	Annexin A8	ANXA8	0.37
48	A Disintegrin and Metalloproteinase domain 10	ADAM10	0.37
49	SLC9A3 Regulator 1	SLC9A3R1	0.36
50	Aldo-Keto Reductase Family 1 Member A1	AKR1A1	0.36
51	14-3-3 Zeta	YWHAZ	0.35
52	Glutathione S-transferase pi 1	GSTP1	0.35
53	Alpha v integrin subunit	ITGAV	0.34
54	Solute carrier family 5 (sodium/myo-inositol cotransporter), member 3	SLC5A3	0.34
55	Solute carrier family 2, facilitated glucose transporter member 1	SLC2A1	0.33

The most enriched proteins in the purest apical RPE-derived exosome preparation identified by protein correlation profiling (PCP) in comparison to a crude apical EV preparation. The pure-to-crude ratio of each protein was normalized to the ratio of Syntenin-1, an exosome-specific marker, and sorted in descending order of pure-to-crude ratio. Proteins co-enriched within a three-fold of Syntenin-1 identified by at least two unique peptides and in two separate experiments are shown. Note the detection by PCP of low abundance proteins among the most highly enriched proteins in the pure exosome preparation (*e*.*g*. SNAP23 and BST1; see “Exosome abundance” tab in Supplemental Tables S1 and S2 for relative abundance data), would likely not be identified as exosome-specific by traditional mass spectrometric analysis which rely only on abundance in individual preparations to rank proteins.

^*^Ratio of the relative abundance in the pure exosome preparation to the relative abundance in the crude EV preparation, normalized to Syntenin-1.

**Table 4:** Proteins enriched in basolaterally released exosomes.

Enrichment ranking	Protein name	Gene name	Pure/Crude*
1	Charged Multivesicular Body Protein 4B	CHMP4B	1.93
2	Annexin A4	ANXA4	1.82
3	SLA-2 histocompatibility antigen, class I	SLA-2	1.70
4	G Protein Subunit Alpha I2	GNAI2	1.69
5	Fibronectin Leucine Rich Transmembrane Protein 2	FLRT2	1.60
6	Actinin Alpha 4	ACTN4	1.58
7	CD2-associated protein	CD2AP	1.42
8	Integrin Subunit Alpha 6	ITGA6	1.14
9	Lactate dehydrogenase A	LDHA	1.13
10	RAB5C, Member RAS Oncogene Family	RAB5C	1.12
11	Annexin A2	ANXA2	1.04
12	Peptidyl-prolyl cis-trans isomerase	PPIA	1.01
13	Syntenin-1	SDCBP	1.00
14	Solute Carrier Family 4 Member 2	SLC4A2	0.96
15	Annexin A5	ANXA5	0.94
16	ALIX	PDCD6IP	0.92
17	Integrin beta 1	ITGB1	0.89
18	A Disintegrin And Metalloproteinase Domain 10	ADAM10	0.85
19	Ezrin	EZR	0.72
20	Annexin A1	ANXA1	0.72
21	CD81	CD81	0.70
22	Heat shock 70 kDa protein 1B	HSPA1B	0.65
23	Clathrin heavy chain	CLTC	0.65
24	Heat Shock Protein 90 kDa Alpha Family Class B Member 1	HSP90AB1	0.63
25	Bestrophin-1	BEST1	0.63
26	Enolase 1	ENO1	0.60
27	Basigin (CD147)	BSG	0.60
28	Hsp70-interacting protein	ST13	0.58
29	Protein Tyrosine Phosphatase, Non-Receptor Type 23	PTPN23	0.55
30	Eukaryotic Translation Elongation Factor 1 Alpha 1	EEF1A	0.54
31	ATPase Na+/K+ Transporting Subunit Alpha 1	ATP1A1	0.52
32	RAB7A, Member RAS Oncogene Family	RAB7A	0.51
33	Stimulated by Retinoic Acid 6	STRA6	0.51
34	Heat shock 70 kDa protein 1A	HSPA1A	0.49
35	Cysteine and Glycine Rich Protein 1	CSRP1	0.49
36	Adenosylhomocysteinase Like 2	AHCYL2	0.46
37	Solute Carrier Family 2 Member 1 (GLUT1)	SLC2A1	0.45
38	EGF Like Repeats and Discoidin Domains 3	EDIL3	0.43
39	Hepatocyte Growth Factor-Regulated Tyrosine Kinase Substrate	HGS	0.42
40	Solute Carrier Family 3 Member 2	SLC3A2	0.42
41	5′-Nucleotidase Ecto	NT5E	0.40
42	Retinaldehyde Binding Protein 1	RLBP1	0.40
43	Heat Shock Protein Family A (Hsp70) Member 2	HSPA2	0.40
44	Vesicle Amine Transport 1	VAT1	0.37
45	Tubulin beta 3	TUBB3	0.36
46	Pyruvate Kinase, Muscle	PKM	0.34
47	Peroxiredoxin 2	PRDX2	0.33

The most enriched proteins in the purest basolateral RPE-derived exosome preparation identified by protein correlation profiling (PCP) in comparison to a crude basolateral EV preparation. Proteins were normalized to the abundance of Syntenin-1, an exosome-specific marker, and sorted by pure-to-crude ratio in descending order. Proteins co-enriched within a three-fold of Syntenin-1 identified by at least two unique peptides in two separate experiments are shown. Note that some of these proteins that are identified as highly enriched in the pure exosome preparation by PCP, are proteins of low abundance (*e*.*g*. FLRT2 and CHMP4B; see “Exosome abundance” tab in Supplemental Tables S3 and S4 for relative abundance data), but are nonetheless unambiguously exosome-specific.

^*^Ratio of the relative abundance in the pure exosome preparation to the relative abundance in the crude EV preparation, normalized to Syntenin-1.

### Directional exosome proteomes

Exosome and small EV preparations, which are inherently heterogeneous mixtures of vesicles, cannot be purified to homogeneity. To overcome limitations of traditional mass spectrometric analysis of these mixtures, we performed mass-spectrometry based PCP analysis^28, 29^ of apically and basolaterally RPE-derived EVs by quantitatively profiling hundreds of proteins in EV preparations of increasing purity.

**Figure 4:**
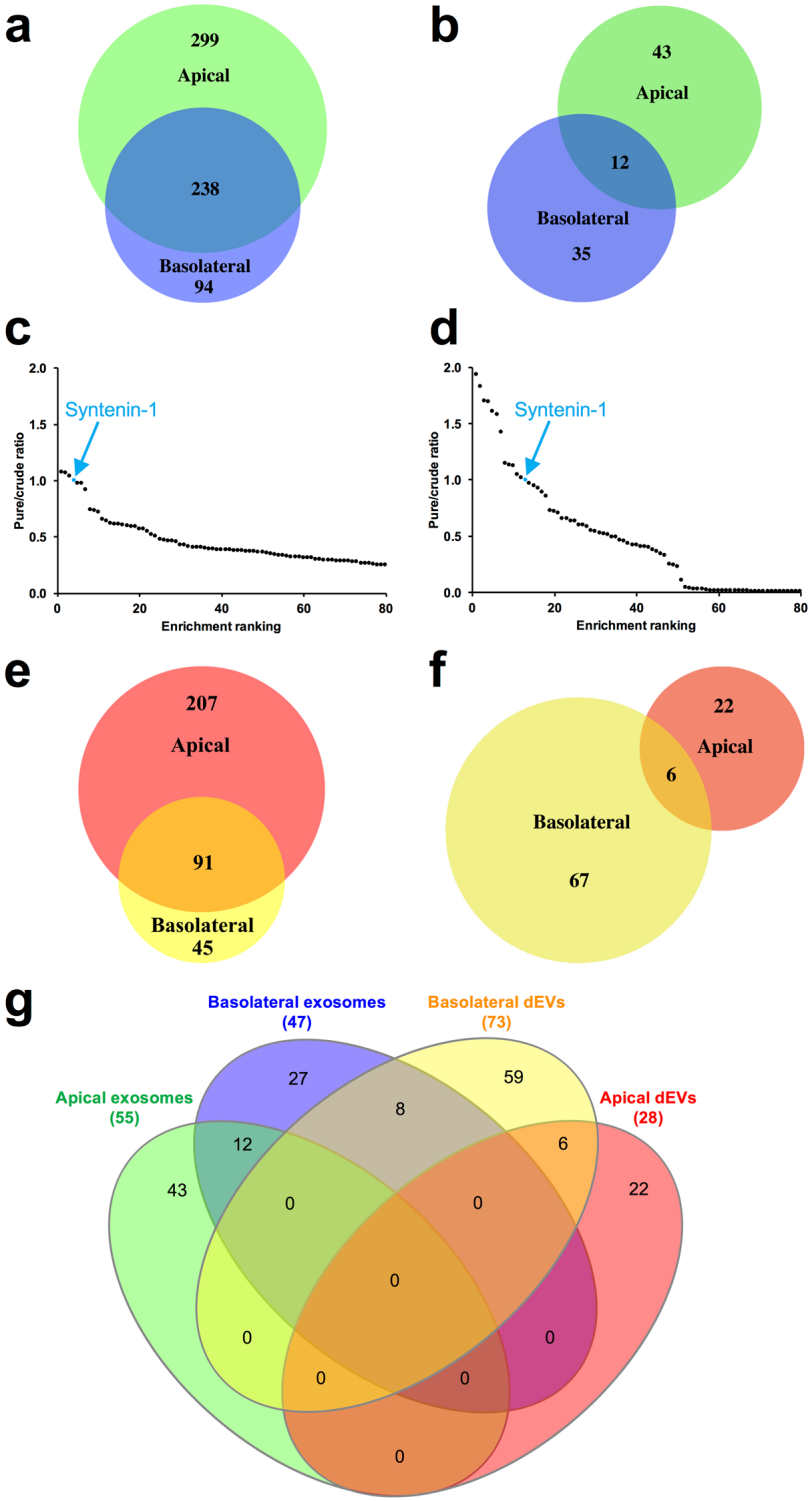
Directional exosome and dense EV (dEV) proteomes shown as Venn diagrams and enrichment ranking graphs. Venn diagrams displaying (**a**) all, and (**b**) proteins within a three-fold enrichment of the exosome marker Syntenin-1, identified with two or more unique peptides in apical (green) and basolateral (blue) exosome preparations from polarized mature RPE monolayers. The identity of the exosomal proteins shown in (**b**) that were bidirectionally released are shown in Table 5. (**c**,**d**) Scatter plots indicating the enrichment ranking of the eighty most enriched proteins in the pure exosome preparation (x-axis) as a function of their pure-to-crude ratio of relative abundance normalized to Syntenin-1 (y-axis). The position of Syntenin-1 is indicated in cyan in the graphs. Panel (**c**) shows apically released, and panel (**d**) basolaterally released exosomal proteins. (**e**,**f**) Venn diagrams of all (**e**), and (**f**) proteins within a three-fold enrichment of the exosome marker Syntenin-1, identified with two or more unique peptides in apical (red) and basolateral (yellow) dEV (fraction 9) preparations from polarized RPE cultures. (**g**) Four-way Venn diagram comparing RPE-derived apical and basolateral exosome and dEV proteomes demonstrate how distinctly different the four EV populations are. The same coloring scheme as seen in panels (**b**) and (**f**), is used in panel (**g**).

A total of 631 different proteins were identified in apical and basolateral exosome preparations, of which 299 were found exclusively in apically secreted exosomes and 94 exclusively in basolaterally released exosomes (Fig. 4a). These distributions highlight the directional difference in protein cargo released in association with exosomes from a polarized cell. Traditionally in these type of PCP analyses, the criterion for robustly co-enriched proteins are those within a two-fold (2.0 to 0.5 ratio values) of the protein used for normalization^29^. However, due to the presence of well-known exosomal proteins such as 5′-Nucleotidase Ecto (NT5E)^35, 36^ outside this range, the proteomes of these exosome preparations appeared more heterogeneous than photoreceptor discs^29^, centrosomes^28^ and mitochondria preparations^37^ analyzed previously by PCP. Thus, we decided to expand this criterion to include proteins enriched within a three-fold (3.0 to 0.33 ratio values) of Syntenin-1, an exosome-specific marker^34^. This criterion is supported by a clear separation of enrichment ratio values in Fig. 4d showing protein enrichment for basolateral exosomes, which was found to be 0.33 for Peroxiredoxin 2 (Table 4) while the next most enriched protein had a ratio of 0.24. When proteins enriched within a three-fold of Syntenin-1 in exosomes released in a directional manner were compared, only 12 of those proteins were present in exosomes from both sides of the RPE monolayer (Fig. 4b, Tables 3–5). The enrichment ranking of the 80 most enriched proteins in the pure exosome preparations were graphed on scatter plots as a function of the protein pure-to-crude ratio of relative abundance normalized to Syntenin-1, an exosome-specific marker^34^ (Fig. 4c,d and Table S5). In exosome preparations isolated from the apical side, 6 proteins co-enriched closely with Syntenin-1 (Fig. 4c, Table 3). In basolateral exosome preparations 10 proteins were enriched to an even higher degree than Syntenin-1 and 7 proteins were closely co-enriched with Syntenin-1 (Fig. 4d and Table 4), indicating a slightly larger heterogeneity in basolateral than apical exosome populations.

**Table 5:** Bidirectionally released proteins found among proteins enriched within a three-fold of Syntenin-1 in apical and basolateral exosome preparations from polarized RPE monolayers.

Protein name	Gene name	Enrichment ranking*	
		Apical	Basolateral
Syntenin-1	SDCBP	4	13
CD81	CD81	1	21
Hsp70-interacting protein	ST13	5	28
ALIX	PDCD6IP	17	16
Heat shock 70 kDa protein 1B	HSPA1B	12	22
Annexin A1	ANXA1	35	20
Annexin A2	ANXA2	46	11
A Disintegrin And Metalloproteinase Domain 10	ADAM10	48	18
Heat Shock Protein Family A (Hsp70) Member 2	HSPA2	26	43
EGF Like Repeats and Discoidin Domains 3	EDIL3	42	38
5′-Nucleotidase Ecto	NT5E	44	41
Solute Carrier Family 2 Member 1 (GLUT1)	SLC2A1	55	37

Proteins are shown in order of the average of enrichment in apical and basolateral exosome preparations.

^*^Enrichment ranking in the pure exosome preparation by descending pure-to-crude ratio of the relative abundance of each protein.

Of the proteins that were uniquely secreted in exosomes on either side, several reflected polarity-specific functions of the RPE monolayer, suggesting that potential biological functions of exosomes released directionally are different. As examples, Bestrophin-1, which is a transmembrane chloride channel with a known basolateral localization, was only found in basolaterally released exosomes (Table 4). Conversely, Solute Carrier Family 39 Member 12 (SLC39A12) also known as ZIP12, is a transmembrane Zinc transporter which was only found in apically released exosomes, [Table 3, Fig. 3e (fractions 6–8)]. The chaperone protein, αB-Crystallin, has previously been shown to be released in association with apical exosomes from polarized RPE cells^15^, and likewise it was only found in the apical exosome proteome (Table 3), further validating the integrity of our polarized *in vitro* RPE model.

Interestingly, the well-established exosome and/or small EV markers CD9 and CD63^34^, were not identified within the three-fold enrichment of Syntenin-1 in basolateral exosomes (Table 4). They both show a much lower enrichment and lower abundance in the basolateral than among apical exosomal proteins, compare Tables S3–S4 with Tables S1–S2, suggesting that basolateral exosomes may be a more heterogeneous population of vesicles than apical exosomes. This is an important finding since many commercial exosome detection and quantification kits rely on the use of antibodies to these two marker proteins.

### Directional dense EV (dEV) proteomes

We also determined the proteomes of apically and basolaterally released dense EVs (dEV) isolated from fraction 9 (ρ = 1.125 g/ml) of our iodixanol gradients (Fig. 4e,f, Tables S6–7). A total of 343 different proteins were identified in apical and basolateral dEV preparations, 207 of which were exclusively found in apically secreted dEVs and 45 exclusively in basolaterally released dEVs (Fig. 4e). Dense EVs have been shown to contain large amounts and a wide range of extracellular matrix (ECM) proteins^34, 38^. The ECM proteoglycan Collagen Type XVIII Alpha 1 (COL18A1 is essential for RPE function and Bruch’s membrane structure)^39^ was highly enriched (Table S10) in both apical and basolateral dEV preparations and was not found in exosomal preparations (Tables S1–S4). Therefore it was chosen as the normalizing protein for enrichment ranking in the dEV datasets. Comparison of proteins enriched within a three-fold of COL18A1 in dEVs released in a directional manner, revealed only 6 of those proteins were present in dEVs from both sides of the RPE monolayer (Fig. 4f, Tables S8–10). Two of the most enriched proteins in the basolaterally released dEVs (Table S9), the complement protein C3 and the amyloid beta precursor protein (APP), are known components of drusen and have been implicated in the AMD disease process^23, 40–42^. Neither of these two proteins were identified in apically released dEVs, or in the exosome proteomes.

A four-way Venn diagram of apical and basolateral exosome and dEV proteomes revealed significant differences in the EV protein composition as a result of both directionality of release and EV subtype (Fig. 4g). Of particular note is the large differences between exosome proteomes and dEV proteomes, highlighting the power of iodixanol gradient centrifugation as part of a PCP analytical approach to resolve distinctly different EV populations.

## Discussion

In order to interrogate the role of EVs in both normal eye biology, and the pathophysiology of AMD and other retinal diseases affecting the RPE, high quality reliable baseline data for physiologically normal RPE-derived EV release is required. To this end, the proteomes of apically and basolaterally released RPE-derived exosomes under homeostatic conditions were analyzed in the current study. In order to ensure that the studied small EV preparations are indeed exosomes, we have adhered to and gone beyond the minimal experimental conditions put forth in a recent position statement of recommendations from the International Society for Extracellular Vesicles (ISEV)^43^.

We used iodixanol buoyancy density gradient ultracentrifugation to isolate EV subpopulations highly enriched for exosomes and small light EVs. Protein Correlation Profiling (PCP) mass spectrometry^28, 29^ was used to compare the relative abundance of proteins identified in the exosome-enriched preparation to that in the crude EV preparation used to load the density gradient. This powerful proteomic analytical approach made it possible to identify proteins as exosome-specific regardless of their abundance in the exosome preparation. This is very difficult to do with a traditional mass spectrometric approach where low abundance proteins are assumed to be contaminants or of minor importance. Thus, our use of PCP provided a heretofore-unequaled fidelity of the apical and basolateral RPE-derived exosomal proteomes. In fact, due to the comparative nature of PCP, absolute purity of a purified preparation is not necessary, only a robust level of enrichment. Since exosomes and other EV preparations are inherently heterogeneous and cannot be purified to homogeneity, PCP represents an ideal method for mass spectrometric analyses of EV preparations. Compared to immunoblotting approaches that only characterize a handful of proteins at a time and is affected by antibody quality, mass spectrometry with the same amount of total protein can identify the entire exosome and/or EV proteome in a preparation without bias. However, as discussed above, the drawback with traditional mass spectrometry is that its high sensitivity and often complicated quantification leads to identification of many proteins which are in fact contaminants but difficult to identify as such. For example, Milk Fat Globule-EGF Factor 8, also known as Lactadherin, is the most abundant protein in many exosome proteomics studies^44^, including in the present study (see the “Exosome abundance” tabs in Tables S1–S4). However, we show in our comparative PCP analysis that it is not specifically enriched into our exosome preparations (see the “Exosome enrichment” tabs in Tables S1–S4). Thus, Lactadherin serves as an example to explain the differences in information gathered by analyzing the *enrichment* into the exosome preparation versus analyzing the *abundance* in the exosome preparation alone. A co-enrichment with a known marker will indicate that the protein in question is strongly associated with exosomes and thus a true exosome-resident protein, while a depletion will suggest a much looser or no attachment/association with exosomes. That is not to say that a protein of low enrichment but high abundance in exosome preparations may not in some cases possess an important biological function when associated with exosomes. Further studies would be needed to identify those proteins in a case-by-case manner. However, in most cases proteins of low enrichment but high abundance in the exosome preparation, are likely to be contaminating proteins. Likewise, proteins of low enrichment and low abundance are unambiguous contaminants.

Using PCP, a total of 631 proteins were profiled in exosome preparations, 299 of which were uniquely released apically, and 94 uniquely released from the basolateral side. One of the highly enriched apical-specific exosomal proteins was the zinc transporter SLC39A12 (ZIP12), which is highly expressed in the eye and in the brain^45^. Interestingly, although SLC39A12 was not identified in the basolateral exosome preparation (fractions 6–8) by PCP or immunoblotting, it was detected in the heavier basolateral fractions 9–12 (ρ = 1.125–1.293 g/ml) (Fig. 3e, Ba panel), suggesting distinctly different cargo sorting into EVs released apically vs basolaterally. Further studies will be needed to clarify the mechanism and potential role of release of these directional-specific EV populations.

An RPE-specific protein which displayed stark directionality was Bestrophin-1, which has a known basolateral localization in polarized RPE^30, 31, 46^ as well as in our pRPE cell cultures (Fig. S3). Accordingly, it was exclusively found in basolaterally released exosomes, indicating the polarization of our RPE monolayers. The proteomes of these exosome preparations suggest that epithelial polarity impacts directional release, resulting in patterns that are consistent with RPE homeostatic functions.

Interestingly, there were 10 proteins which were enriched to an even higher degree than Syntenin-1 in the basolaterally released exosomes (Fig. 4d). This suggests that the population of light EVs isolated from basolateral media are more heterogeneous than the more apparently classical exosome population isolated from apical media which display relatively high enrichment of the three traditional tetraspanin exosome markers CD81, CD9 and CD63. That said, Syntenin-1 positive EVs from basolateral media equilibrated at classical exosome densities (fractions 6–8; ρ = 1.074–1.108 g/ml)^33, 34^, while apical Syntenin-1 EVs also appeared (albeit at lower amounts) to equilibrate at lower densities (fractions 3–5) in addition to the classical exosome densities (fractions 6–8) (Fig. 3c). Further studies will be needed to characterize the very light Syntenin-1 positive apical EVs in fractions 3–5. The basolaterally released exosomes, which display a somewhat different proteome than traditional exosomes, may need to be further sub-fractionated and/or purified to clarify whether novel exosome-like vesicles are responsible for the observed proteomic differences.

Although the RPE has vastly different functions at its apical and basal surfaces *in vivo*, which is also reflected in the EVs secreted in either direction; only apically released EVs can be obtained from *ex vivo* eyecups^32^. In contrast, the *in vitro* RPE model we have presented here can be used to study both apical and basolateral vesicle release and to test how disease-associated stressors affect it. This is important because the pathognomonic drusen and sub-RPE deposits in AMD involve primarily basolateral nutrient (carbohydrates, amino acids etc.), lipoprotein particle and EV transport in their formation^7, 47^. Thus, it is of crucial importance to study the basolateral in addition to apical release of EVs. Supporting the polarity-specific functions of *in vivo* RPE, our study demonstrates that an *in vitro* polarized, differentiated RPE culture model has distinct apical and basolateral exosome proteomes. Exemplifying this, as discussed above, the zinc transporter SCL39A12^45^ was found in apically released exosomes, while the basolaterally located chloride channel Bestrophin-1^46^ was found in basolaterally secreted exosomes. Disruptions in the function of Bestrophin-1 causes a range of macular dystrophies, most notably Vitelliform Macular Dystrophy or Best’s disease^48^. Interestingly, SLC39A12 was recently shown to play a major role in cellular responses to hypoxia^49^, which has been proposed to be one of the mechanisms of RPE stress in AMD and other retinal diseases^50^. It will be interesting to investigate in further studies whether exosomal release of these two proteins modulate disease mechanisms in any way.

There are very few previous studies that have investigated the role of directionally released exosomes and small EVs from polarized RPE cell cultures by studying both apical and basolateral release^15, 51, 52^, and none has taken an unbiased global approach to characterize directional EV release. Sreekumar and colleagues^15^ showed that the neuroprotective chaperone αB-crystallin is released in association with exosomes only apically under homeostatic conditions. Another study showed that shRNA knockdown of αB-Crystallin inhibits both apical and basolateral exosome release^51^, potentially implicating αB-Crystallin in the endocytic sorting machinery and EV formation within RPE cells. Singh and co-workers^52^ recently showed that RPE derived from pluripotent stem cells obtained from Best disease patients displayed increased exosome release both apically and basolaterally, suggesting a potential role of exosomes in certain pathological conditions. Wang and colleagues showed by immunohistological methods, the presence of proteins that may be in exosomes and other EVs, in sub-RPE areas in mouse tissues and human postmortem eyes^19, 20, 53^. In the present study, we report that known protein components of drusen and other sub-RPE deposits such as complement C3, APP and a number of ECM proteins^54–57^, were predominantly found in basolaterally secreted dEVs rather than exosomes (compare Tables 4 and S9). A portion of these dEVs may be ectosomes, vesicles defined by their shedding from the plasma membrane as opposed to the MVE-released exosomes, and their larger size and density compared to exosomes^11^. Thus, these findings support a role of ectosome release in the formation of drusen and other sub-RPE deposits, although extensive further studies are needed to support this idea.

In a recent study characterizing exosomes, dEVs and a range of other EVs, it was suggested that complement C3, and PEDF among other proteins found in association with dEVs probably come from the serum added to cultures and associate extracellularly with EVs after their secretion^34^. Since our studies were carried out under serum-free culturing conditions, all of the proteins identified in our dEV preparations, that are traditionally thought to come from serum (*e*.*g*, C3 and fibronectin), are in fact expressed by the RPE cells themselves.

Interestingly, a recent study showed that under serum-free conditions of exosome collection from polarized ARPE-19 (spontaneously immortalized) cultures, the total exosome release was about two-fold higher on the apical versus basolateral side^51^. We show similar results in the present study under FBS-supplemented culture conditions (Table 1). However, under culture conditions using the serum supplement B-27 instead of FBS, exosome release is shifted to close to equal apical and basolateral release (Table 1). The potential role or significance of this finding is unclear at present but does potentially highlight another difference between ARPE-19 cell cultures and primary RPE cultures. Further studies using different culturing conditions and/or known stressors will be needed to study potential changes in RPE-derived EV release.

A noteworthy finding in our directional exosome proteomes was that the well-established exosome and/or small EV markers CD9 and CD63^34^, were significantly more enriched in the apical exosome proteome (Tables 3, S1–S2) compared to the basolateral proteome (Tables 4, S3–S4). This is an important finding that has implications for the broader field of EV research since many exosome detection and quantification kits rely on the use of antibodies to these two marker proteins. Thus, it appears that care should be taken to characterize the protein content of directionally released EVs to ensure that appropriate markers are used. Similar to our results using PCP, a recent study using immunoblotting-based detection, found that CD63 was found exclusively in exosomes released apically from polarized epithelial cells^12^. It is unclear at present if the lower enrichment of CD9 in basolaterally released exosomes is specific for RPE or includes all polarized epithelia, and if the mostly apical-specific release of CD63 is a hallmark of all polarized epithelia or even other polarized cell types.

In summary, to our knowledge this is the first study defining apical and basolateral EV and/or exosome proteomes in a differentiated RPE model. Specifically, we identified the normal apical and basolateral exosome proteomes by an in-depth mass spectrometric approach that identified the most highly enriched proteins in RPE-derived exosomes. This analysis lays the foundation for future comparative proteomic studies of apically and basolaterally released exosomes and other EVs; and for a more comprehensive understanding of mechanisms underlying diseases affecting the RPE. Future studies investigating changes in the directional exosome proteome in response to stressors relevant to AMD and other retinal diseases will enhance our understanding of, and suggest targeted therapies toward, disease-specific mechanisms. In addition to providing insight into pathophysiological processes and thereby identifying potential drug targets for retinal diseases, RPE-derived exosome proteomic findings may also offer potential biomarkers for prognostic, diagnostic or therapeutic use. Exosomes are uniquely amenable to biomarker analysis because of their relatively high stability in body tissues and fluids^58^. Exosome isolation from eye fluids such as tear fluid and aqueous humor, or systemic circulation (plasma, serum) and urine, could be analyzed for RPE-specific protein (or nucleic acid) markers identified in the current study. These RPE-specific markers could be used for additional immunopurification steps and/or as diagnostic indicators of retinal disease.

## Methods

### Antibodies and reagents

Calcium and magnesium free PBS (PBS^−^) was purchased from Gibco (#10010-023). Triton X-100 was obtained from Sigma-Aldrich (#T8787). Hoechst 33258 (#H3569) and AlexaFluor 568-conjugated Phalloidin (#A12380) were from Invitrogen. Antibodies used were as follows: Mouse anti-RPE65 (#ab78036 [clone 401.8B11.3D9]; Abcam, Cambridge, MA), mouse anti-Cytokeratin (#M0821 [clone MNF116]; Dako A/S, Glostrup, Denmark), rabbit anti-ZO1 (mid) (#40-2200; ThermoFisher Scientific, Waltham, MA), mouse anti-Syntenin-1 (#ab131190 [clone 3D9-G9-H4]; Abcam), mouse anti-TSG101 (#612696; BD Biosciences, San Jose, CA), rabbit anti-SLC39A12 (#ab106570; Abcam), rabbit anti-Calreticulin (#12238 [clone D3E6]; Cell Signaling Technologies, Danvers, MA), mouse anti-BEST1 (#NB300-164, [clone E6-6]; Novus Biologicals, Littleton, CO), mouse anti-Na^+^/K^+^-ATPase alpha (#sc-58628 [clone M7-PB-E9]; Santa Cruz Biotechnology, Dallas, TX), rabbit anti-CD36 (#ab78054; Abcam), AlexaFluor 488-conjugated donkey-anti-rabbit IgG (#A21206, Invitrogen), AlexaFluor 488-conjugated donkey-anti-mouse IgG (#A21202, Invitrogen), AlexaFluor 568-conjugated donkey-anti-rabbit IgG (#A10042; Invitrogen), HRP-conjugated donkey-anti-mouse IgG (#715-035-150, Jackson ImmunoResearch Laboratories, West Grove, PA), and HRP-conjugated donkey-anti-rabbit IgG (#711-035-152, Jackson ImmunoResearch Laboratories).

### Polarized RPE cell culture

Primary cultures of porcine RPE cells were prepared according to a previously published protocol^31^ with minor modifications. Briefly, porcine eyes were trimmed of excess tissue and anterior segments (including the entire lens and vitreous) were removed with a scalpel at the ora serrata. Eyecups (posterior poles) were placed into individual wells of 6-well tissue culture cluster plates (Corning #3516). Eyecups were filled with 2 ml of PBS containing 1 mM EDTA (pre-warmed to 37 °C) and incubated in a 37 °C 5% CO_2_ incubator for 30 min to loosen the retina. After removal of the retina, eyecups were filled with 2 ml of 0.25% trypsin-100 mM EDTA solution (Gibco #25200-056) and placed in an incubator for 30 min. RPE cells were recovered by repeated aspiration followed by a low speed centrifugation (5 min at 300 *g*). Cells were seeded at 60% confluence on 75 cm^2^ cell culture flasks (Corning #430641) and allowed to grow to >90% confluence before being trypsinized for seeding onto cell culture inserts. Thus, cells were seeded at passage p1 onto Laminin/Entactin (Corning #354259) coated 24 mm cell culture inserts with pore sizes of 0.4, 1.0, or 3.0 µm. Inserts (0.4 and 3.0 µm were from Corning (Transwell™, #3450 and 3452) while 1.0 µm inserts were from Greiner Bio-One (ThinCert™, #657610). Cells were seeded at high density onto inserts (300,000 RPE cells/cm^2^), see ref. 31. Under these high density seeding conditions, 100% confluence is achieved immediately upon seeding. RPE cells on inserts were maintained in DMEM with glucose and sodium pyruvate (Gibco #11995-065) supplemented with 1% (v/v) heat-inactivated FBS (Mediatech #35-010-CV), 50 units/ml penicillin, 50 ug/ml streptomycin, 100 mM L-glutamine (Sigma #G6784), 0.25 µg/ml Amphotericin B (Gibco #15290-018), and 10 µg/ml ciprofloxacin (Corning #61-277-RF). This medium will be referred to as *complete pRPE medium* with FBS. The same complete pRPE medium was supplemented with B-27 serum supplement for certain experiments as described below. In both culture conditions, high levels of pigmentation was achieved after as little as 1–2 weeks on cell culture inserts, with B-27 cultures displaying higher pigmentation than FBS cultures with increasing culture time.

### Immunofluorescence microscopy

Porcine RPE monolayers on cell culture inserts were fixed with 4% paraformaldehyde in DMEM for 15 min at 20 °C. Membranes were excised from the insert cups and cut into pieces for in-solution staining in 2 ml microcentrifuge tubes (#1620-2700, USA Scientific Inc., Ocala, FL). RPE cell monolayers were blocked and permeabilized for 30 min in 10% normal donkey serum (NDS; #017-000-121, Jackson Immunoresearch, West Grove, PA), 0.5% Triton X-100 in PBS^−^. Membranes were incubated overnight at 4 °C with primary antibodies diluted in 1% NDS, 0.1% Triton X-100 in PBS^−^. Primary antibodies used were diluted as follows: rabbit anti-bovine RPE65 1:200, anti-Cytokeratin 1:10, rabbit anti-ZO1 1:100, mouse anti-BEST1 1:200, mouse anti-Na^+^/K^+^-ATPase 1:100, rabbit anti-CD36 1:200. Membranes were washed thrice for 5 min each with PBS^−^ and incubated for 2 hrs at room temperature with AlexaFluor 488-conjugated anti-rabbit IgG or anti-mouse IgG antibodies diluted 1:500, or AlexaFluor568-conjugated anti-rabbit IgG antibodies diluted 1:500, AlexaFluor 568-conjugated Phalloidin (1:100) and the nuclear stain Hoechst 33258 (1:500) in the same buffer used for primary antibodies. Filters were finally washed four times for 10 min each with PBS^−^ and mounted on glass slides (Superfrost; #12-550-15, Fisher Scientific) with ProLong Diamond Antifade mounting medium (#P36965, Invitrogen), coverslipped (LifterSlips; #22X22I24788001LS, ThermoFisher Scientific) and sealed with clear nail polish (#72180; Electron Microscopy Sciences, Hatfield, PA). Cells were imaged with a Nikon Eclipse 90i confocal microscope (Nikon Instruments, Melville, NY).

### Transepithelial electrical resistance (TEER)

Transepithelial electrical resistance (TEER), which is inversely proportional to the paracellular permeability of cultured RPE cells and is a reliable assay for the assessment of RPE barrier function^59–62^, was measured by means of a volt-ohm meter equipped with a 24-mm EndOhm chamber (World Precision Instruments, Sarasota, FL). Resistance values for each condition were determined from a minimum of three individual cultures and corrected for the inherent cell culture insert resistance within five minutes after removing the plates from the incubator. All values represent the mean ± S.E.M.

### Conditioned media for EV isolation

For generation of conditioned media for EV isolation, cell media was exchanged with media supplemented with EV-depleted FBS or with B-27 serum supplement (#17504044, Gibco). Mass spectrometry was performed on B-27 supplement to confirm that unknown exogenous proteins were not present. Only the defined B-27 component proteins were found (serum albumin, catalase, insulin, transferrin, superoxide dismutase). To avoid contamination with FBS-derived EVs from the complete pRPE medium, cells were cultured for two days in the EV collection media after which it was discarded and fresh collection media was added. EV-depleted FBS was prepared as described previously^33^. Briefly, 20% (v/v) FBS was centrifuged in a Beckman LE-80K ultracentrifuge using an SW-28 rotor at 100,000 *g* for 18 hrs, 4 °C. The supernatant was carefully collected without disturbing the loose pellet and sterile-filtered through a 0.22 µm PVDF filter (Millipore), aliquoted and frozen at −20 °C until used.

For EV collection from cell culture inserts: Conditioned media from four to six 6-well cluster plates with 24 mm permeable inserts were collected daily for three to four weeks. The volumes used were 1.5 ml in the upper (apical) chamber and 2.6 ml in the lower (basal) chamber for each insert. For an average exosome preparation, 300–400 ml of apical and 500–700 ml of basal conditioned media was used as starting material.

For EV collection from porcine eyecups: Six eyecups with the retina removed were rinsed twice in warmed complete pRPE medium, then 1.5 ml complete pRPE medium with B-27 growth supplement was added to each eyecup and they were placed in a humidified incubator at 37 °C, 5% CO_2_ for 25 min.

### EV isolation

Two experimental protocols were used to isolate EVs:

#### EV isolation by differential centrifugation

EVs were isolated using a modification of a well-established differential centrifugation protocol as previously described^33, 63^. Briefly, conditioned medium was centrifuged at 2,000 *g* for 10 min to remove cell debris and the resulting supernatant was collected and kept at −80 °C until the next steps of the isolation protocol. Cleared conditioned media was centrifuged at 10,000 *g* for 30 min and the resulting supernatant was transferred to a new tube and centrifuged at 100,000 *g* for 90 min. The resulting supernatant was discarded and the EV pellet was resuspended in PBS^−^ from a freshly opened bottle to wash away contaminants and centrifuged again at 100,000 *g* for 90 min. The EV pellet was resuspended in a lysis buffer (2% SDS, 100 mM Tris-HCl [pH 6.8], 10 mM DTT) if the sample was prepared for mass spectrometry and immunoblotting analyses, or resuspended in PBS^−^ if prepared for NTA analysis and electron microscopy. Centrifugations at 10,000 *g* (*k*-factor = 2547.2) and 100,000 *g* (*k*-factor = 254.7) were done at 4 °C using polyallomer tubes (Beckman Coulter Inc., Indianapolis, IN; #326823) in an SW-28 rotor in a Beckman LE-80 K ultracentrifuge (Beckman Coulter).

#### EV isolation by iodixanol buoyant density gradient centrifugation

Conditioned media previously cleared at 2,000 *g* was concentrated using Centricon Plus-70 centrifugal filter devices with 100 kDa NMWL cutoff (Millipore, #UFC710008). The concentrated media was recovered and made up to 37 ml with PBS^−^, placed in a polyallomer tube and centrifuged at 100,000 *g* in an SW-28 rotor for 90 min. The resulting crude EV pellet was resuspended in 1.1 ml PBS^−^, 1.0 ml of which was collected and used for subsequent density gradient centrifugation. The remaining 0.1 ml of the EV pellet was diluted to 37 ml with PBS- in the same tube and washed by an additional centrifugation at 100,000 *g* for 90 min. The resulting pellet was lysed in 50 μl 2% SDS, 100 mM Tris-HCl [pH 6.8], 10 mM DTT; designated *crude EV pellet*, and used for mass spectrometry and immunoblot analyses. 1.0 ml of the resuspended EV pellet was mixed with 2 ml of a 60% (w/v) iodixanol solution (OptiPrep™; Sigma-Aldrich #D1556) to make up the bottom 40% fraction. A discontinuous gradient of iodixanol solutions was prepared by carefully overlaying the bottom fraction with 3 ml of 20%, 10%, and 5% solutions of iodixanol buffered with 0.25 M sucrose, 10 mM Tris-HCl (pH 7.5), respectively. The gradient tubes (UltraClear™; Beckman Coulter #344059) were subjected to centrifugation at 100,000 *g* (SW-41 rotor; 28,500 rpm) for 18 h at 4 °C. One ml fractions were collected manually from the top of the self-generated gradient and weighed to determine density. One ml fractions were diluted 12-fold with PBS^−^ and subjected to centrifugation at 100,000 *g* for 90 min in the SW41 rotor. Pellets were resuspended in 25 μl 2% SDS, 100 mM Tris-HCl [pH 6.8], 10 mM DTT and stored at −80 °C until use. Total protein content in EV preparations were determined with the Pierce 660 nm protein assay (ThermoFisher Scientific) using a NanoDrop-2000 instrument (ThermoFisher Scientific).

### Immunoblotting

Western blot analysis was performed as previously described^41^. Briefly, cell lysate, crude EV and purified iodixanol gradient fraction samples were run reduced, on 10% Bis-Tris Criterion XT gels in MOPS buffer, transferred to nitrocellulose, and then probed with indicated antibodies. Anti-Syntenin-1, anti-TSG101, anti-SLC39A12, and anti-Calreticulin were all used at 1:1,000 dilutions. Subsequent incubation with horseradish peroxidase–conjugated secondary antibodies at 1:12,500 dilution was followed by detection with ECL Plus reagent (Pierce Biotechnology). ECL signals and total protein loading amounts were measured with a Bio-Rad ChemiDoc MP imaging system (Bio-Rad Laboratories Inc., Hercules, CA). The acquired images were analyzed with Bio-Rad ImageLab software (Bio-Rad Laboratories).

### Transmission electron microscopy (TEM)

EV samples for TEM analysis were prepared according to a previously published protocol^33^. Briefly, EV preparations in PBS^−^ were fixed in 2% paraformaldehyde and deposited on formvar coated copper grids (#01702-F; TED Pella Inc., Redding, CA). Grids were negatively stained with a uranyl oxalate solution followed by a positive stain with a uranyl acetate-methylcellulose solution. Images were collected using a JEOL JEM-1400 electron microscope at 60 kV.

### Proteomic sample preparation

EV samples in lysis buffer (2% SDS, 100 mM Tris-HCl [pH 6.8], 10 mM DTT) from two to four separate EV isolations were pooled and prepared for proteomic analysis and digested with trypsin using a recently reported paramagnetic bead-based protocol^64^. Two separate pooled exosome preparations (two biological repeats) and one pooled dEV preparation of apical and basolateral origin, respectively, were analyzed by mass spectrometry. The sensitivity and integrity of the preparation method was validated by analyzing samples of varying amounts of porcine RPE cell lysates which had been subjected to the protocol (data not shown). This bead-based protocol provided a more comprehensive proteomic identification than gel-based protocols^64^.

### Mass spectrometry analysis

Tryptic peptides eluated from the beads were vacuum-dried and dissolved in 2% acetonitrile, 0.25% formic acid. Peptides (typically 0.5-1 µg) were analyzed using a nanoAcquity UPLC system coupled to a Synapt G2 HDMS mass spectrometer (Waters Inc.) employing LC-MS/MS experiment in a data independent acquisition mode complemented with ion mobility separation (HDMSE). Samples were analysed in duplicates on 1.7 mm 75 mm × 150 mm C18 130 A BEH column (Waters Inc.) using a 90 min 5% to 30% gradient of acetonitrile in 0.1% formic acid at a flow rate 0.3 ml/min at 35 °C. Eluting peptides were sprayed into the ion source of the Synapt G2 using the 10 μm PicoTip emitter (Waters Inc.) at a voltage of 2.5 kV.

### Label-free protein identification and quantification using Progenesis QI for Proteomics

For a protein quantification experiment, duplicate data-independent analyses (HDMSE) for each sample were conducted in similar LC settings for simultaneous peptide identification and quantification. For robust peak detection and alignment of individual peptides across all HDMSE runs we performed automatic alignment of ion chromatography peaks representing the same mass/retention time features using Progenesis QI software. To perform peptide assignment to the features, PLGS 2.5.1 (Waters Inc.) was used to generate searchable files that were submitted to the IdentityE search engine incorporated into Progenesis QI for Proteomics. For peptide identification, we searched against the UniProt *Sus Scrofa* protein database (July 2016 release) using Cys carbamidomethyl as constant modification and Met oxidation as variable. Protein abundances in crude EV preparations and the highly exosome-enriched density gradient purified fractions were calculated from the sum of all unique peptide ion intensities normalized by the factor that makes the same value for the total intensity of all peptides in all experimental samples. Conflicting peptides for different proteins and their isoforms were excluded from the calculations. For protein identification in cultured RPE cell lysates (Supplemental Table S11), we used a similar HPLC, acquisition and Progenesis analysis method but omitted the ion peak area measurements.

### Protein correlation profiling

The relative quantities of proteins across all EV and exosome preparations were normalized to abundance of the well-established exosome marker Syntenin-1 (SDCBP)^34^. All identified proteins were ranked based on their abundance ratios between the enriched purified preparation and the crude EV preparation that was used to generate the highly exosome-enriched preparations (Tables 3–5, S1–S4). PCP analyses of dEV-enriched (fraction 9) preparations (Tables S6–10) were normalized to COL18A1. The area-proportional Venn diagrams of exosome and dEV proteomes in Fig. 4a,b and e,f, were generated using the web-based BioVenn tool^65^. The four-way Venn diagram in Fig. 4g was generated using the web-based InteractiVenn tool^66^.

### Nanoparticle tracking analysis

Nanoparticle tracking analysis (NTA) was performed using a NanoSight NS500 Instrument (Malvern Instruments Inc., Westborough, MA) equipped with a 532 nm laser and integrated automated fluidics. Five 60-second videos were recorded of each sample with camera level set at 14 and detection threshold set at 6. Temperature was set at 23 °C and monitored throughout the measurements. Videos recorded for each sample were analyzed with NTA software version 3.0 to determine the concentration and size of measured particles with corresponding standard error. For analysis, auto settings were used for blur, minimum track length and minimum expected particle size. The NanoSight system was calibrated with polystyrene latex microbeads of 50, 100, and 200 nm (Thermo Scientific Inc.) prior to analysis. PBS^−^ (Gibco #10010-023) was used as diluent and to avoid contaminating particles a fresh bottle was opened for each analysis session. At least three separate EV preparations for each condition were analyzed.

### Statistical analysis

TEER measurements, concentrations of small EVs released from RPE, and modal and mean EV sizes, were tested for statistical significance using a two-sample two-tailed Student’s t-test assuming unequal variance. P-values < 0.05 were considered statistically significant.

## Electronic supplementary material

Supplementary information

Supplementary Dataset 1

Supplementary Dataset 2

Supplementary Dataset 3

Supplementary Dataset 4

Supplementary Dataset 5

Supplementary Dataset 6

Supplementary Dataset 7

Supplementary Dataset 11
