# Author Correction: Directional Exosome Proteomes Reflect Polarity-Specific Functions in Retinal Pigmented Epithelium Monolayers

**DOI:** 10.1038/s41598-018-35542-w

**Published:** 2018-11-20

**Authors:** Mikael Klingeborn, W. Michael Dismuke, Nikolai P. Skiba, Una Kelly, W. Daniel Stamer, Catherine Bowes Rickman

**Affiliations:** 1 Department of Ophthalmology, Duke Eye Center, Duke University, Durham, NC 27710 USA; 2 Department of Biomedical Engineering, Duke University, Durham, NC 27710 USA; 3 Department of Cell Biology, Duke University, Durham, NC 27710 USA

Correction to: *Scientific Reports* 10.1038/s41598-017-05102-9, published online 07 July 2017

This Article contains typographical errors in the Acknowledgements section.

“This study was supported by a BrightFocus Foundation grant M2015221 (MK), NIH grants EY023468 (WMD), EY0123456 (CBR), EY023287 (WDS), EY022359 (WDS), EY019696 (WDS), a Glaucoma Research Foundation Shaffer Grant (WMD, WDS), and a grant from the Foundation Fighting Blindness (CBR). A Core Grant for Vision Research (P30; EY5722) from NEI (to Duke University), supported much of the work, including the mass spectrometric analyses carried out by NPS.”

should read:

“This study was supported by a BrightFocus Foundation grant M2015221 (MK), NIH grants EY023468 (WMD), EY026161 (CBR), EY023287 (WDS), EY022359 (WDS), EY019696 (WDS), a Glaucoma Research Foundation Shaffer Grant (WMD, WDS), and a grant from the Foundation Fighting Blindness (CBR). A Core Grant for Vision Research (P30; EY5722) from NEI (to Duke University), supported much of the work, including the mass spectrometric analyses carried out by NPS.”

