# Supplementary material for: Directional Exosome Proteomes Reflect Polarity-Specific Functions in Retinal Pigmented Epithelium Monolayers
Source: Sci Rep. 2017 Jul 7;7:4901. doi: 10.1038/s41598-017-05102-9 (PMC5501811; doi:10.1038/s41598-017-05102-9)
Supplement: Supplementary file 1 — Supplementary information [file 41598_2017_5102_MOESM1_ESM.doc]

**Supplementary Information**

**Directional Exosome Proteomes Reflect Polarity-Specific Functions**

**in Retinal Pigmented Epithelium Monolayers**

Mikael Klingeborn^1^,*, W. Michael Dismuke^1^, Nikolai P. Skiba^1^, Una Kelly^1^, W. Daniel Stamer^1,2^ and Catherine Bowes Rickman^1,3^,*

^1^Department of Ophthalmology, Duke Eye Center, Duke University, Durham, NC 27710

^2^Department of Biomedical Engineering, Duke University, Durham, NC 27710

^3^Department of Cell Biology, Duke University, Durham, NC 27710

^*^Corresponding authors: Mikael Klingeborn, Duke Eye Center, DUMC Box 3802, AERI Rm 5040, Durham, NC 27710; Phone: 919-668-0649, E-mail: [mikael.klingeborn@duke.edu](mailto:mikael.klingeborn@duke.edu); Catherine Bowes Rickman, Duke Eye Center, DUMC Box 3802, AERI Rm 5010, Durham, NC 27710; Phone: 919-668-0648, E-mail: [bowes007@duke.edu](mailto:bowes007@duke.edu).

**Supplemental Tables S1 and S2. Excel files containing lists of all the proteins identified in two separate apical exosome preparations with pure-to crude ratio values and abundance values.** Proteins sorted according to enrichment into the exosome preparation are shown in the tab “Exosome enrichment” and proteins sorted according to relative abundance in the exosome preparation are shown in the tab “Exosome abundance”.

**Supplemental Tables S3 and S4.** **Excel files containing lists of all the proteins identified in two separate basolateral exosome preparations with pure-to crude ratio values and abundance values.** Proteins sorted according to enrichment into the exosome preparation are shown in the tab “Exosome enrichment” and proteins sorted according to relative abundance in the exosome preparation are shown in the tab “Exosome abundance”.

**Supplemental Table S5.** **Excel file containing the 80 most enriched proteins identified in apical and basolateral exosome preparations including measures of the coefficients of variation of the data.** Proteins sorted according to the aggregate enrichment in the two datasets for apical and basolateral exosome preparations are shown with the standard variation and coefficient of variation for each protein in the two datasets.

**Supplemental Tables S6 and S7.** **Excel files containing lists of all the proteins identified in apical (S6) and basolateral (S7) dense EV preparations with pure-to crude ratio values and abundance values.** Proteins sorted according to enrichment into the dEV preparation are shown in the tab “Enrichment” and proteins sorted according to relative abundance in the dEV preparation are shown in the tab “Abundance”.

**Supplemental Table S8.** **Proteins enriched in apically released dense EVs.** The most enriched proteins in the purest apical RPE-derived dEV preparation (fraction 9) identified by protein correlation profiling (PCP) in comparison to a crude apical EV preparation. Proteins were normalized to the abundance of COL18A1, and sorted by fraction 9-to-crude ratio in descending order. Proteins co-enriched within a three-fold of COL18A1 identified by at least two unique peptides are shown.

| **Enrichment ranking** | **Protein name** | **Gene name** | **Pure/Crude*** |
| --- | --- | --- | --- |
| 1 | Prolyl 4-Hydroxylase Subunit Beta | P4HB | 1.14 |
| 2 | Collagen Type XVIII Alpha 1 Chain | COL18A1 | 1.00 |
| 3 | Pyruvate Dehydrogenase (Lipoamide) Beta | PDHB | 0.95 |
| 4 | Collagen Type XIV Alpha 1 Chain | COL14A1 | 0.94 |
| 5 | Galectin 3 Binding Protein | LGALS3BP | 0.82 |
| 6 | Angiopoietin-like protein 7 | ANGPTL7 | 0.75 |
| 7 | Ferritin | FTL | 0.69 |
| 8 | Heat Shock Protein 90 Beta Family Member 1 (GP96) | HSP90B1 | 0.67 |
| 9 | Glycogen synthase 1 | GYS1 | 0.66 |
| 10 | Heat Shock Protein Family A (Hsp70) Member 9 | HSPA9 | 0.60 |
| 11 | Ribophorin I | RPN1 | 0.59 |
| 12 | Tenascin | TNC | 0.57 |
| 13 | Malate dehydrogenase | MDH2 | 0.56 |
| 14 | Isocitrate Dehydrogenase (NADP(+)) 2, Mitochondrial | IDH2 | 0.54 |
| 15 | ADAMTS Like 5 | ADAMTSL5 | 0.53 |
| 16 | Collagen Type I Alpha 2 Chain | COL1A2 | 0.52 |
| 17 | Atlastin GTPase 3 | ATL3 | 0.48 |
| 18 | Slit Guidance Ligand 3 | SLIT3 | 0.47 |
| 19 | ATRX, Chromatin Remodeler | ATRX | 0.46 |
| 20 | Collagen Type V Alpha 1 Chain | COL5A1 | 0.44 |
| 21 | Nucleolin | NCL | 0.42 |
| 22 | Collagen Type I Alpha 1 Chain | COL1A1 | 0.41 |
| 23 | Major Vault Protein | MVP | 0.40 |
| 24 | Slit Guidance Ligand 2 | SLIT2 | 0.37 |
| 25 | Heparan Sulfate Proteoglycan 2 (Perlecan) | HSPG2 | 0.35 |
| 26 | Fibronectin | FN1 | 0.34 |
| 27 | Nidogen 2 | NID2 | 0.33 |
| 28 | Collagen Type XI Alpha 1 Chain | COL11A1 | 0.33 |

*Ratio of the relative abundance in the dEV preparation to the relative abundance in the crude EV preparation, normalized to COL18A1.

**Supplemental Table S9.** **Proteins enriched in basolaterally released dense EVs.** The most enriched proteins in the purest basolateral RPE-derived dEV preparation (fraction 9) identified by protein correlation profiling (PCP) in comparison to a crude basolateral EV preparation. Proteins were normalized to the abundance of COL18A1, and sorted by fraction 9-to-crude ratio in descending order. Proteins co-enriched within a three-fold of COL18A1 identified by at least two unique peptides are shown.

| **Enrichment ranking*** | **Protein name** | **Gene name** | **Pure/Crude^†^** |
| --- | --- | --- | --- |
| 2 | C-type lectin domain family 18 member A | CLEC18A | 1.92 |
| 3 | Retinol Dehydrogenase 5 | RDH5 | 1.72 |
| 4 | Annexin A4 | ANXA4 | 1.07 |
| 5 | ATP synthase subunit alpha, mitochondrial | ATP5A1 | 1.00 |
| 6 | Collagen Type XVIII Alpha 1 Chain | COL18A1 | 1.00 |
| 7 | Complement C3 | C3 | 0.91 |
| 8 | ADAMTS Like 5 | ADAMTSL5 | 0.86 |
| 9 | ATP synthase subunit beta, mitochondrial | ATP5B | 0.84 |
| 10 | Growth Arrest Specific 6 | GAS6 | 0.79 |
| 11 | Keratin 71 | KRT71 | 0.76 |
| 12 | Amyloid Beta Precursor Protein | APP | 0.75 |
| 13 | Actinin Alpha 4 | ACTN4 | 0.69 |
| 14 | Acyl-CoA Synthetase Short-Chain Family Member 3 | ACSS3 | 0.68 |
| 15 | Alpha-2-Macroglobulin | A2M | 0.67 |
| 16 | Ornithine carbamoyltransferase, mitochondrial | OTC | 0.66 |
| 17 | Keratin 25 | KRT25 | 0.64 |
| 18 | Aldehyde dehydrogenase family 1 subfamily A3 | ALDH1A3 | 0.64 |
| 19 | Tubulin Alpha 1c | TUBA1C | 0.62 |
| 20 | Myosin Heavy Chain 9 | MYH9 | 0.59 |
| 21 | Sodium/potassium-transporting ATPase subunit beta | ATP1B1 | 0.56 |
| 22 | Cardiac muscle alpha actin 1 | ACTC1 | 0.56 |
| 23 | Solute carrier family 25 member 31 | SLC25A31 | 0.56 |
| 24 | Phosphoglucomutase 1 | PGM1 | 0.54 |
| 25 | Glutaryl-CoA dehydrogenase, mitochondrial | GCDH | 0.52 |
| 26 | Propionyl-CoA carboxylase alpha chain, mitochondrial | PCCA | 0.52 |
| 27 | Thrombospondin 3 | THBS3 | 0.52 |
| 28 | Collagen Type V Alpha 1 Chain | COL5A1 | 0.51 |
| 29 | MHC class I antigen | SLA-3 | 0.51 |
| 30 | Arginase-1 | ARG1 | 0.51 |
| 31 | Lactate Dehydrogenase A | LDHA | 0.50 |
| 32 | Heparan Sulfate Proteoglycan 2 (Perlecan) | HSPG2 | 0.49 |
| 33 | Glycogen phosphorylase, liver form | PYGL | 0.49 |
| 34 | Phosphoglycerate kinase 1 | PGK1 | 0.48 |
| 35 | Isocitrate Dehydrogenase (NADP(+)) 2, Mitochondrial | IDH2 | 0.48 |
| 36 | Aldehyde Dehydrogenase 1 Family Member A1 | ALDH1A1 | 0.47 |
| 37 | Isocitrate dehydrogenase [NADP] | IDH1 | 0.47 |
| 38 | Argininosuccinate Lyase | ASL | 0.47 |
| 39 | Aldehyde Dehydrogenase 7 Family Member A1 | ALDH7A1 | 0.47 |
| 40 | Aldehyde dehydrogenase family 8 member A1 | ALDH8A1 | 0.46 |
| 41 | Glutamate dehydrogenase 1, mitochondrial | GLUD1 | 0.46 |
| 42 | Epoxide hydrolase 2 | EPHX2 | 0.45 |
| 43 | Actin Gamma 1 | ACTG1 | 0.45 |
| 44 | Hemoglobin subunit alpha | HBA1 | 0.44 |
| 45 | HtrA Serine Peptidase 1 | HTRA1 | 0.44 |
| 46 | Ketohexokinase | KHK | 0.44 |
| 47 | 2,4-Dienoyl-CoA Reductase 1, Mitochondrial | DECR | 0.43 |
| 48 | Serpin Family E Member 2 | SERPINE2 | 0.42 |
| 49 | Centrosomal protein 250kDa | CEP250 | 0.42 |
| 50 | Annexin A5 | ANXA5 | 0.42 |
| 51 | Tubulin Beta 4B Class IVb | TUBB4B | 0.41 |
| 52 | Betaine--Homocysteine S-Methyltransferase | BHMT | 0.41 |
| 53 | Acetyl-CoA Acyltransferase 1 | ACAA1 | 0.41 |
| 54 | Short-chain-specific acyl-CoA dehydrogenase, mitochondrial | ACADS | 0.40 |
| 55 | Annexin A6 | ANXA6 | 0.40 |
| 56 | Heat Shock Protein Family A (Hsp70) Member 5 | HSPA5 | 0.40 |
| 57 | Mitochondrial aldehyde dehydrogenase 2 | ALDH2 | 0.40 |
| 58 | Beta-Ureidopropionase 1 | UPB1 | 0.40 |
| 59 | Elongation factor 1-alpha | EEF1A | 0.39 |
| 60 | Aldehyde Oxidase 1 | AOX1 | 0.39 |
| 61 | Carbamoyl-Phosphate Synthase 1 | CPS1 | 0.39 |
| 62 | Heat Shock Protein 90 Alpha Family Class B Member 1 | HSP90AB1 | 0.39 |
| 63 | Dihydropyrimidinase | DPYS | 0.39 |
| 64 | Fumarylacetoacetate Hydrolase | FAH | 0.38 |
| 65 | UDP-glucose 6-dehydrogenase | UGDH | 0.38 |
| 66 | Collagen Type VIII Alpha 2 Chain | COL8A2 | 0.37 |
| 67 | Integrin beta 1 | ITGB1 | 0.37 |
| 68 | Transferrin | TF | 0.37 |
| 69 | Collagen Type VIII Alpha 1 Chain | COL8A1 | 0.35 |
| 70 | ATPase Na+/K+ Transporting Subunit Alpha 1 | ATP1A1 | 0.35 |
| 71 | Ubiquitin B | UBB | 0.34 |
| 72 | ATPase H+/K+ Transporting Non-Gastric Alpha2 Subunit | ATP12A | 0.34 |
| 73 | Fibulin 2 | FBLN2 | 0.34 |

*Fibronectin was the most enriched protein in the basolateral fraction 9 preparation. However, the enrichment ratio appeared to be abnormally high (see Table S10), which prompted us to not exclude it from this table.

^†^Ratio of the relative abundance in the dEV preparation to the relative abundance in the crude EV preparation, normalized to COL18A1.

**Supplemental Table S10.** **Bidirectionally released proteins found among the 50 most enriched proteins in apical and basolateral dEV preparations (fraction 9) from polarized RPE monolayers.** Proteins are shown in order of the average of enrichment in apical and basolateral dEV preparations.

|  |  | **Enrichment ranking*** | |
| --- | --- | --- | --- |
| **Protein name** | **Gene name** | **Apical** | **Basolateral** |
| Collagen Type XVIII Alpha 1 Chain | COL18A1 | 2 | 6 |
| ADAMTS Like 5 | ADAMTSL5 | 15 | 8 |
| Fibronectin | FN1 | 26 | 1 |
| Collagen Type V Alpha 1 Chain | COL5A1 | 20 | 28 |
| Isocitrate Dehydrogenase (NADP(+)) 2, Mitochondrial | IDH2 | 14 | 35 |
| Heparan Sulfate Proteoglycan 2 (Perlecan) | HSPG2 | 25 | 32 |

*Enrichment ranking in the dEV preparation by descending fraction 9-to-crude ratio of the relative abundance of each protein.

**Supplemental Table S11.** **Excel file containing all proteins identified by mass spectrometry in lysate of transwell cultures of differentiated RPE cells.** Proteins sorted according to number of unique peptides identified for each protein. Proteins uniquely expressed by differentiated RPE cells are highlighted in yellow.

**
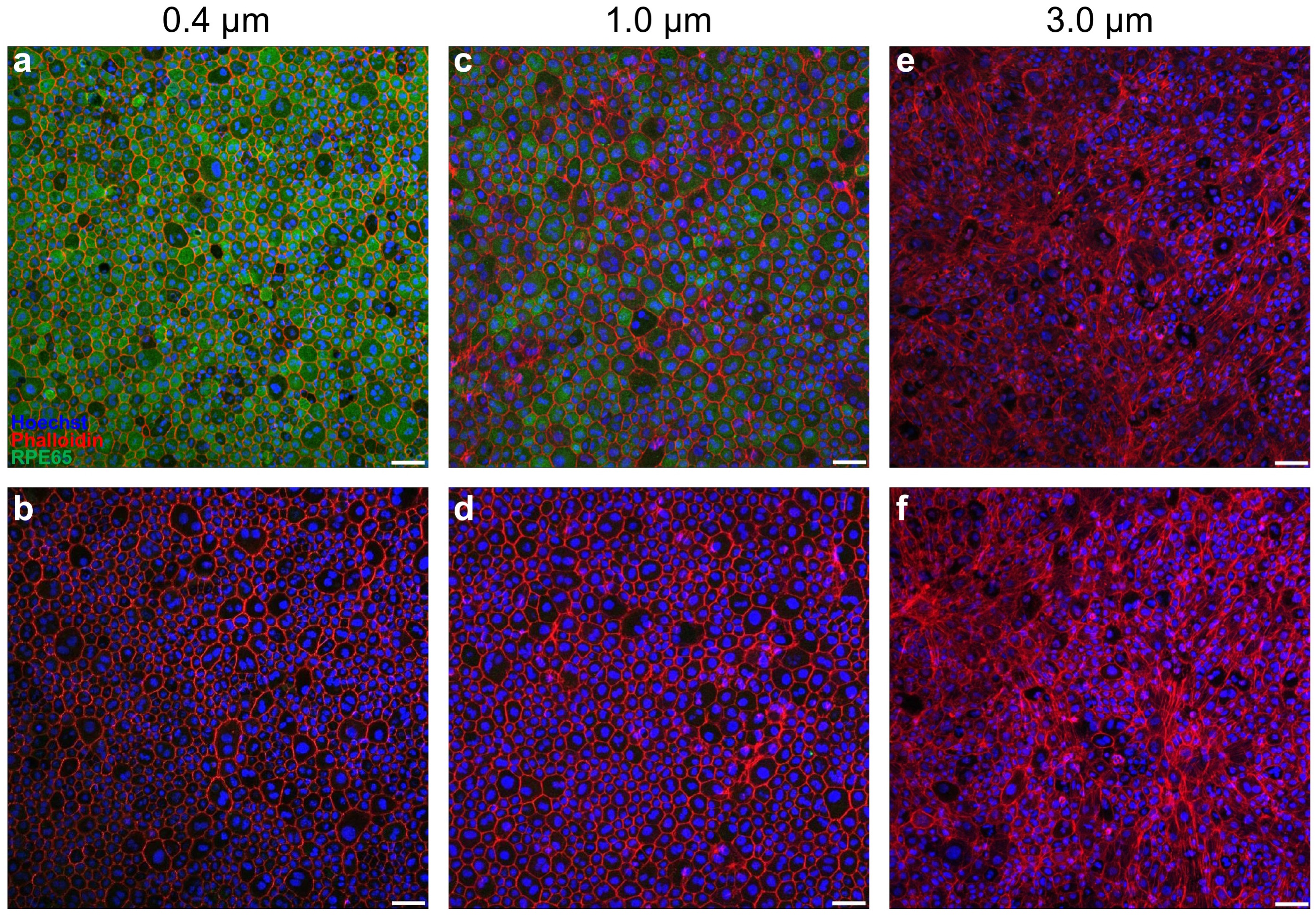
**

**Supplemental Figure S1.** **Morphology of primary porcine RPE (pRPE) monolayers grown on inserts of varying pore size.** pRPE cultures grown on cell culture inserts with 0.4 (**a**-**b**), 1.0 (**c**-**d**) and 3.0 µm (**e**-**f**). Phalloidin (red) stains F-actin along cell borders, displaying the hexagonal cell shape. The widespread cytosolic staining for the RPE-specific marker RPE65 (green) in cultures grown on 0.4 µm pore inserts indicates highly differentiated RPE cells, see panel **a**. Antibody to RPE65 was omitted in panels **b**, **d** and **f**. In RPE monolayers grown on 1.0 and 3.0 μm filters, phalloidin staining shows actin stress fibers (panels **d** & **f**), and lower or lacking RPE65 staining indicates non-differentiated cells (**c** and **e**). Nuclear Hoechst staining is in blue. Scale bars are 50 µm.

**
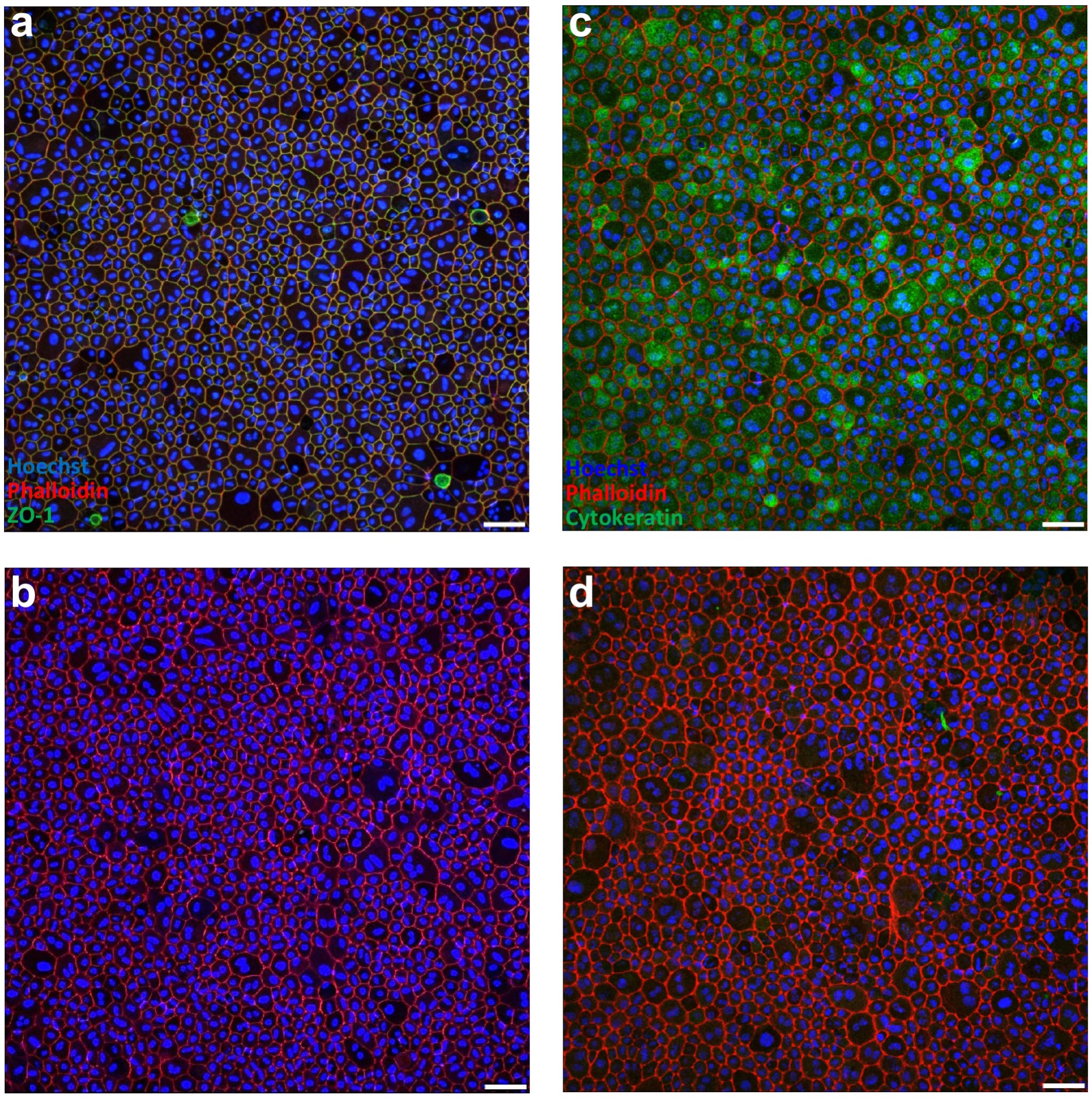
**

**Supplemental Figure S2.** **Primary pRPE cultures grown on cell culture inserts with 0.4 µm pore size.** (**a**) Staining of F-actin with phalloidin (red) along cell borders display hexagonal cell shape and staining for the tight junction-associated protein ZO-1 (green) overlaps with the phalloidin staining and appears yellow. The antibody against ZO-1 was omitted in (**b**). (**c**) Widespread cytosolic staining for the epithelial cell-specific marker cytokeratin (green) indicates that these cells are epithelial cells. Antibody to cytokeratin was omitted in (**d**). Nuclear Hoechst staining is in blue. Scale bars are 50 µm.


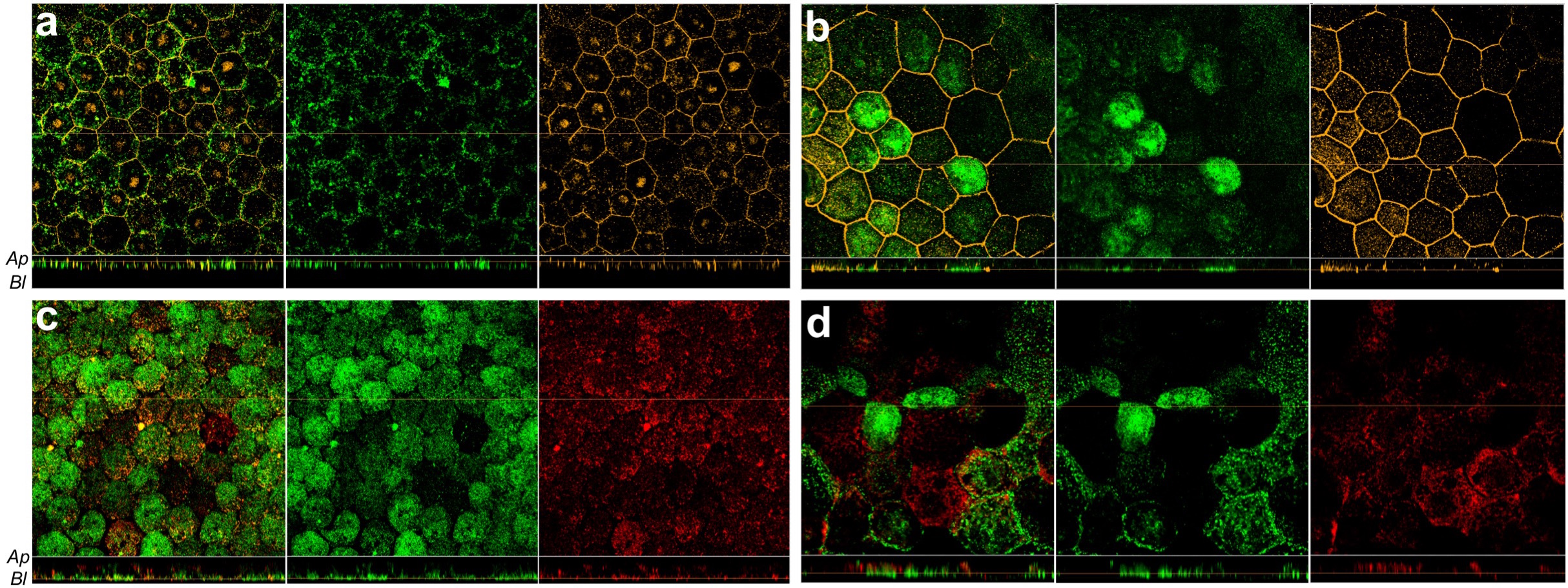


**Supplemental Figure S3.** Confocal immunofluoresecence staining in porcine RPE-choroid punches (dia=6mm) (**a** and **c**), and porcine RPE transwell cultures (**b** and **d**), stained for Na^+^/K^+^-ATPase alpha (green) and ZO-1 (orange) in **a** and **b**; Bestrophin-1 (green) and CD36 (red) in **c** and **d**. In each panel the leftmost image is a merge of the separate channels shown to the right. *En face* (x-y) views of optical section (z slice) close to the apical (**a** and **b**) and basal (**c** and **d**) RPE plasma membrane are shown, respectively. (**a-d**) Location of the optical section is indicated with a yellow line in the x-z view (lower panels). Location of the x-z view (z stack) is in turn indicated with a yellow line on the *en face* view (upper panels). (**a** and **b**) Na^+^/K^+^-ATPase alpha staining is apical in both porcine RPE-choroid and porcine RPE transwell cultures. (**c** and **d**) The majority of Bestrophin-1 staining is localized to the basal membrane. In addition, some lateral (side) Bestrophin-1 staining localized along cell borders (*en face* view in **c** and **d**) accounted for the majority of the apparent staining in the apical portion of the x-z view (lower panels in **c** and **d**). CD36 is found localized both apically and basolaterally. Z-stack is composed of 33 and 31 (**a-b**) or 33 (**c-d**) optical sections spaced 0.25 µm apart and imaged using a 100X oil immersion objective. Ap = Apical, Bl = Basal.

**
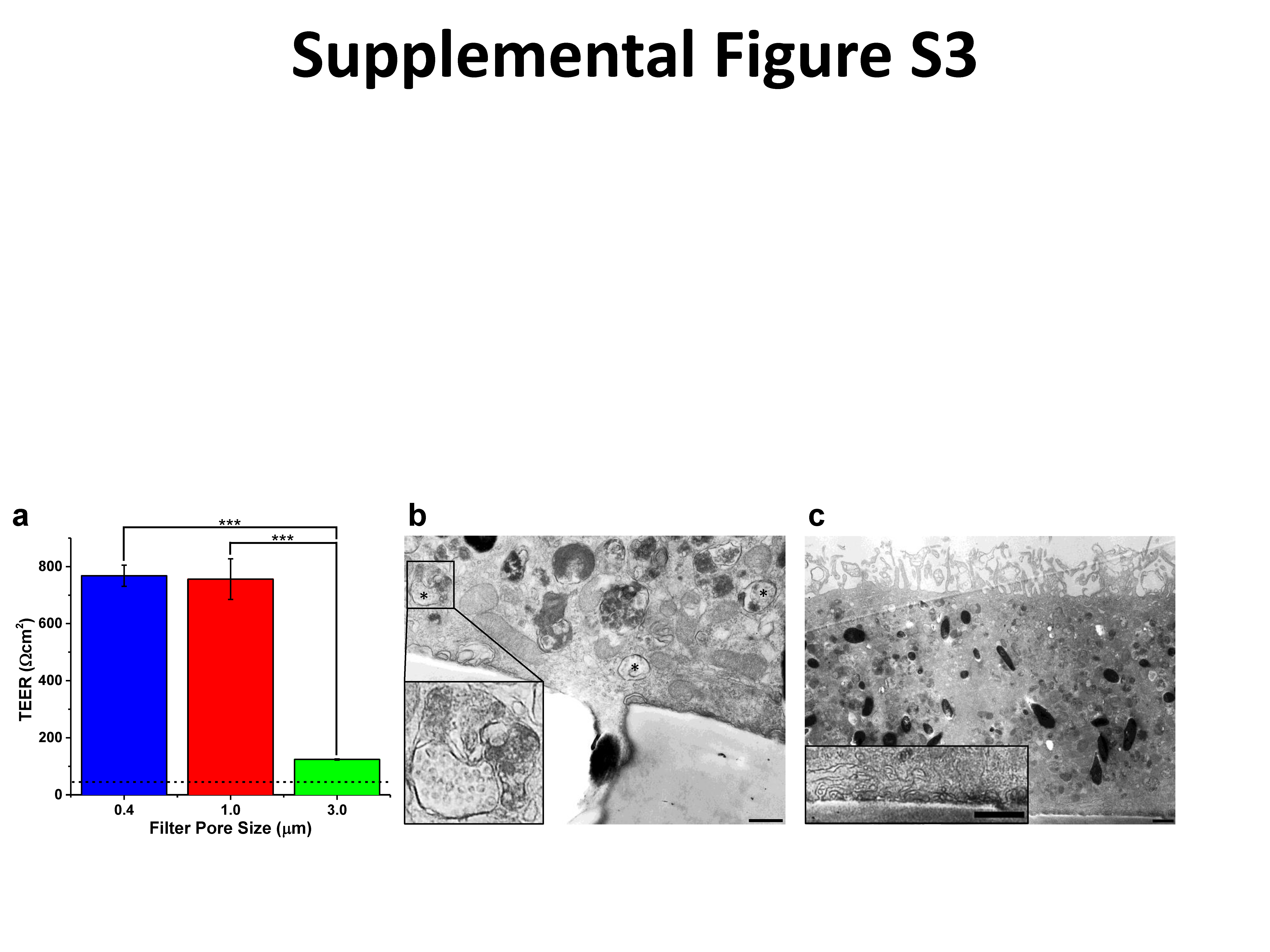
**

**Supplemental Figure S4.** **Effects of cell culture insert pore size on barrier function and ultrastructure of pRPE cultures.** (**a**) TEER was measured in 3.5-wk-old primary pRPE monolayers grown on cell culture inserts with different pore sizes. SEM is indicated, three to fourteen replicates per pore size. Dashed line indicates representative transepithelial electrical resistance (TEER) (43±5 Ωcm^2^) in ARPE-19 cultures on 0.4 µm cell culture inserts^1^. *** p < 0.001. (**b-c**) Electron micrographs of porcine RPE cells grown on 1.0 µm pore cell culture insert (**b**) Area close to the basolateral membrane is shown. Asterisks indicate multivesicular endosomes (MVEs) containing vesicles with sizes corresponding to exosomes. Inset shows one of the MVEs at higher magnification where the individual vesicles can be seen more clearly. (**c**) Apical microvilli and basal infoldings (inset) can clearly be seen, indicating terminal differentiation of RPE cells in these cultures. Inset shows several basal infoldings at higher magnification of the area in the lower right corner of the image. Scale bars are 500 nm (**b**) and 1 µm (**c**), respectively.

**
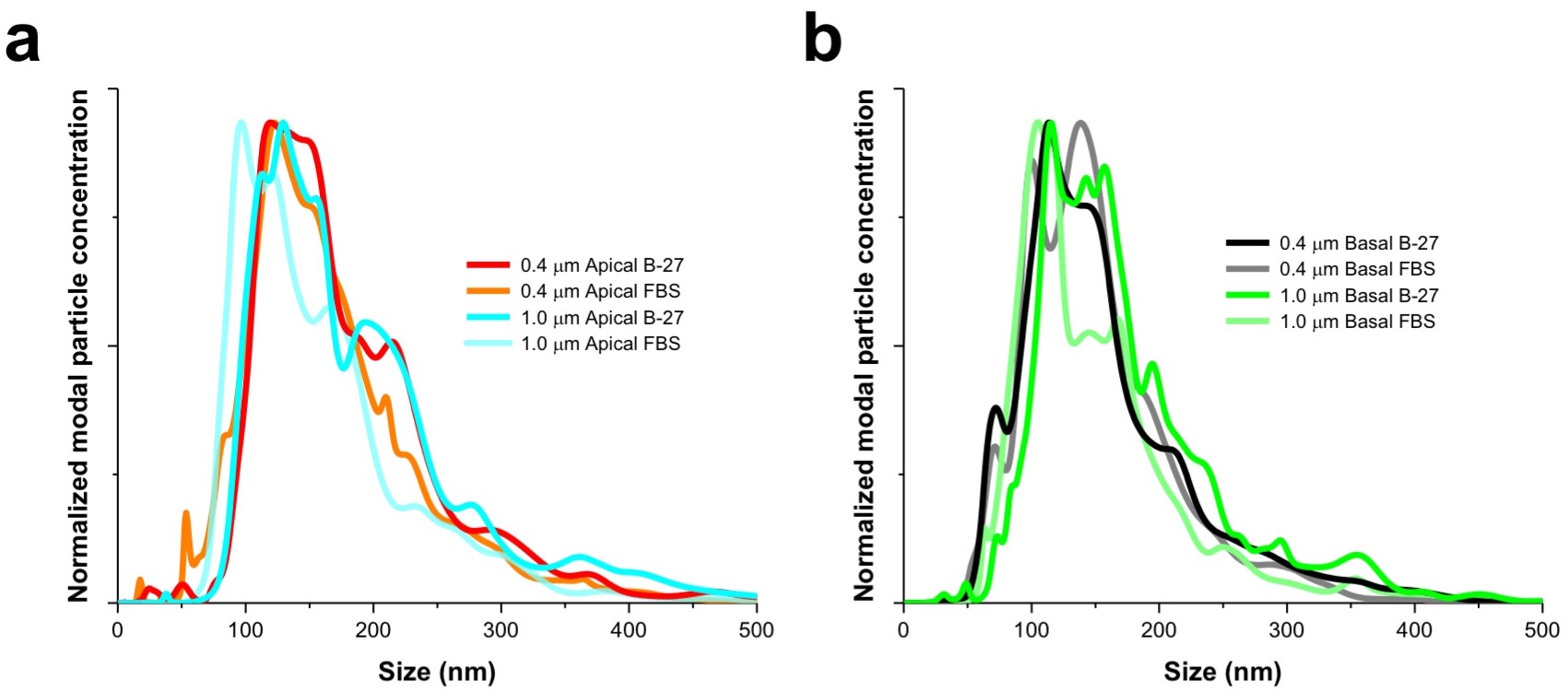
**

**Supplemental Figure S5.** **Size distribution of EVs released from polarized RPE monolayers.** (**a**) EVs released apically from polarized RPE grown on cell culture inserts with 0.4 µm (red and cyan traces) and 1.0 µm pore sizes (orange and light cyan traces). EVs released under B-27- (red and orange) and FBS-supplemented culture conditions (cyan and light cyan) are indicated. Note that the modal particle size is very similar between all of the apically released EVs. (**b**) EVs released basolaterally from polarized RPE grown on cell culture inserts with 0.4 µm (black and green traces) and 1.0 µm pore sizes (gray and light green traces). EVs released under B-27- (black and gray) and FBS-supplemented culture conditions (green and light green) are indicated. Note that the modal particle size is very similar between all of the basolaterally released EVs. Size distributions displayed are averages of three or more separate experiments.

**References**

1 Ablonczy, *Z. et a*l. Human retinal pigment epithelium cells as functional models for the RPE in vivo*. Invest Ophthalmol Vis S*c**i** 52, 8614-8620, doi:10.1167/iovs.11-8021 (2011).
